# Modulating Water Splitting Kinetics via Charge Transfer and Interfacial Hydrogen Spillover Effect for Robust Hydrogen Evolution Catalysis in Alkaline Media

**DOI:** 10.1002/advs.202302358

**Published:** 2023-06-23

**Authors:** Yiming Jiang, Juncai Leng, Shiqi Zhang, Tingyi Zhou, Mingxuan Liu, Shuoming Liu, Yahui Gao, Jianwei Zhao, Lei Yang, Li Li, Wei Zhao

**Affiliations:** ^1^ State Key Laboratory of Food Science and Resources School of Food Science and Technology Jiangnan University Wuxi Jiangsu 214122 P. R. China; ^2^ Shenzhen HUASUAN Technology Co. Ltd. Shenzhen 518055 P. R. China

**Keywords:** heterostructure, hydrogen evolution reaction, hydrogen spillover effect, interfacial electron transfer, water dissociation

## Abstract

Designing and synthesizing advanced electrocatalysts with superior intrinsic activity toward hydrogen evolution reaction (HER) in alkaline media is critical for the hydrogen economy. Herein, a novel Ir@Rhene heterojunction electrocatalyst is synthesized via epitaxially confining ultrasmall and low‐coordinate Ir nanoclusters on the ultrathin Rh metallene accompanying the formation of Ir/IrO_2_ Janus nanoparticles. The as‐prepared heterojunctions display outstanding alkaline HER activity, with an overpotential of only 17 mV at 10 mA cm^−2^ and an ultralow Tafel slope of 14.7 mV dec^−1^. Both structural characterizations and theoretical calculations demonstrate that the Ir@Rhene heterointerfaces induce charge density redistribution, resulting in the increment of the electron density around the O atoms in the IrO_2_ site and thus delivering much lower water dissociation energy. In addition, the dual‐site synergetic effects between IrO_2_ and Ir/Rh interface trigger and improve the interfacial hydrogen spillover, thereby subtly avoiding the steric blocking of the active site and eventually accelerating the alkaline HER kinetics.

## Graphical abstract

A novel Ir@Rhene heterostructure is demonstrated to exhibit outstanding catalytic activity toward alkaline hydrogen evolution reaction by causing charge density redistribution and triggering hydrogen spillover effects along the direction from the metallic oxide to the alloy interface. This work presents a new strategy for fabricating advanced electrocatalysts with all favorable features and offers a fresh perspective on customizing electrocatalytic performance.

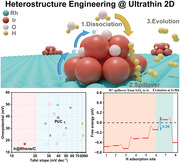

## 1 Introduction

Exploiting high‐performance electrocatalysts toward the hydrogen evolution reaction (HER) is motivated by the urgency in realizing a hydrogen economy in modern society.^[^ ^1^ ^]^ The alkaline HER is favored in industrial applications benefiting from its mild working conditions.^[^ ^2^ ^]^ However, alkaline HER must first undergo a sluggish water dissociation step to produce protons, resulting in 2–3 orders of magnitude lower catalytic efficiency than acidic HER in which protons are more accessible.^[^ ^3^ ^]^ Based on the classic volcano theory, rhodium (Rh) or Rh‐based catalysts are among the most promising candidates for alkaline HER.^[^ ^4^ ^]^ Unfortunately, the relatively high hydrogen adsorption free energy on the Rh metal surface causes the slow kinetics of hydrogen desorption.^[^ ^5^ ^]^ This inefficiency in the subsequent water‐splitting process motivates efforts toward tuning electronic structures of electrocatalysts to enhance the efficacy of electrocatalysts for hydrogen evolution.

Among various reliable strategies for tuning the electronic structure and electron transfer pathway of electrocatalysts, interfacial engineering based on heterostructures is the most widespread and effective.^[^ ^6^ ^]^ In this strategy, the strong electronic interaction at the heterostructured interfaces could generate electronic charge density redistribution and establish electronic communication, optimizing the adsorption behavior of reaction intermediates.^[^ ^7^ ^]^ Nevertheless, the monotonic electrons/mass transfer at the interface of most catalyst architecture fails to simultaneously optimize the catalyst electronic structure for balancing hydrogen adsorption/desorption behaviors and avoiding active site blocking.^[^ ^8^ ^]^ Therefore, exploiting advanced catalyst architecture is critical for the optimal whole water electrolysis kinetics.

Herein, we proposed a simple heterostructure engineering strategy to prepare the ultrathin Ir@Rh metallene (Ir@Rhene) electrocatalysts toward efficient hydrogen evolution in alkaline media. The Ir@Rhene heterostructures are fabricated by epitaxially confining the low‐coordinate and sub‐nm Ir nanoclusters on the Rh metallene (Rhene) accompanying the formation of Ir/IrO_2_ Janus nanoparticles. We demonstrate that the presence of Ir/IrO_2_ Janus nanoclusters causes charge density redistribution and thus modulates water adsorption behavior, accompanying much lower dissociation energy. Meanwhile, the interfacial hydrogen spillover from IrO_2_ to Ir/Rh interface site avoids the steric blocking of the active site and accelerates the whole water‐splitting process, thus exhibiting an HER overpotential of outstanding 17 mV at a current density of 10 mA cm^−2^ in alkaline electrolyte, an ultralow Tafel slop of 14.7 mV dec^−1^, and a superior mass activity of 4.61 A mg_Ir+Rh_^−1^ at 50 mV.

## 2 Results and Discussion

### 2.1 Fabrication and Characterization of Ir@Rhene

**Figure 1:**
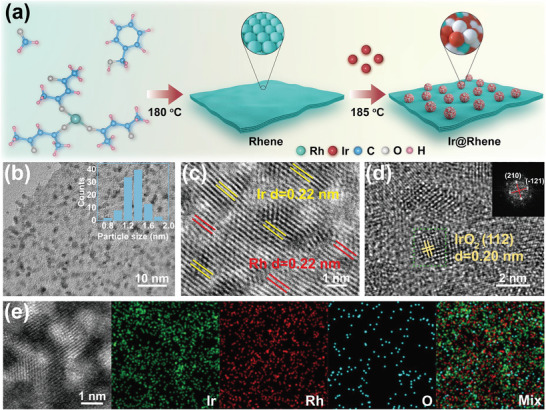
Characterizations of Ir@Rhenes heterostructure. a) Schematic diagram of the synthesis method, b) typical low‐magnification TEM image (inset: size distribution of the nanocluster), c) typical HRTEM image, d) aberration‐corrected HRTEM image and corresponding selected‐area FFT pattern, and e) HADDF‐STEM‐EDS elemental mapping images of Ir@Rhene heterostructure.

As illustrated in **Figure** **1**a, the fabrication procedures of the Ir@Rhene heterostructures involved two major steps, including the synthesis of ultrathin Rh metallene (denoted as Rhene) via reducing Rh(acac)_3_ precursor in formaldehyde/benzyl alcohol solution at 180 °C,^[^ ^9^ ^]^ and the deposition of Ir nanoclusters on the Rhene by a wet‐chemical approach (see the Experimental Section for details). Scanning electron microscopy (SEM), transmission electron microscopy (TEM), high‐resolution TEM (HRTEM), and atomic force microscopy (AFM) images reveal that the Rhene possesses an obvious ultrathin 2D graphene‐like structures and are characterized by lateral dimensions in the range of a few hundred nanometers, lattice spacings of 0.22 nm (corresponding to the Rh (111) facets), and an average thickness of 1.9 nm (Figure S1, Supporting Information). After depositing Ir nanoclusters, the morphology of metallene preserves the initial ultrathin nanosheet structure (Figure S2a–c, Supporting Information). Figure 1b indicates that the Ir nanoclusters with an average diameter of around 1.42 nm are uniformly dispersed on the surface of Rhene. In addition, the thickness and the Ir content of Ir@Rhene heterostructure were measured to be about 2.7 nm and 40.86 wt%, respectively (Figure S2d–h, Supporting Information). The HRTEM and aberration‐corrected HRTEM were further carried out to investigate structural information of the Ir@Rhene at the atomic scale. As illustrated in Figure 1c, the lattice spacings of 0.22 nm can be assigned to the (111) facets of either face‐centered cubic (*fcc*) Ir or Rh. The X‐ray diffraction (XRD) pattern (Figure S3, Supporting Information) also confirms that the Ir@Rhene heterostructure shows a typical *fcc* structure (JCPDS Nos. 05‐0685 and 87‐0715). The crystal lattice with a lattice spacing of 0.20 nm and an angle of 90° was further measured, corresponding to the (112) facets of IrO_2_, as demonstrated by Figure 1d. More importantly, high‐angle annular dark‐field scanning TEM (HADDF‐STEM) energy‐dispersive X‐ray spectroscopy (EDS) further precisely verifies the existence of partially oxidized of Ir atoms (Figure 1e).These findings demonstrate the co‐existence of the zero‐valent and high‐valent state Ir ions, representing superior catalytic potential toward HER because of the accelerating water dissociation by the partially oxidized surface of the Ir cluster.^[^ ^10^ ^]^ Based on these initial results, we conclude that the Ir@Rhene heterostructure comprises an ultrathin Rh metallene and the sub‐nm Ir/IrO_2_ Janus nanoclusters.

**Figure 2:**
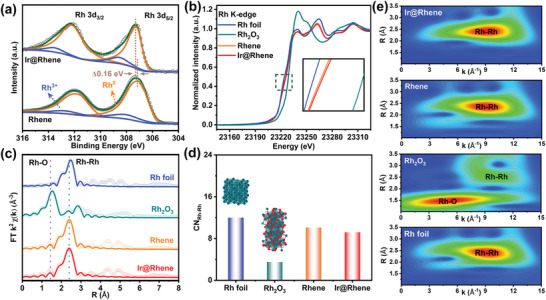
X‐ray absorption characterizations of Rh component in Ir@Rhenes heterostructure. a) Rh 3d XPS spectrum, b) XANES spectrum at the Rh K‐edge, c) Fourier‐transformed (FT) of *k* ^2^‐weighted *χ*(*k*)‐function of the EXAFS spectrum, d) coordination numbers of various catalysts, and e) wavelet transform spectra of Ir@Rhene, Rhene, Rh_2_O_3_, and Rh foil, respectively.

We subsequently employ X‐ray photoelectron spectroscopy (XPS) to characterize the chemical state and elements valence of the as‐prepared heterostructure. The corresponding survey spectra clearly display that the sample mainly consists of three elements: Ir, Rh, and O (Figure S4, Supporting Information). **Figure** **2**a shows the high‐resolution Rh 3*d* XPS spectrum for Ir@Rhene and Rhene, both metallic and oxidized states of Rh species are concomitant, in which the metallic Rh is the dominant phase. In the XPS spectra, interestingly, the peak positions for the Ir@Rhene spectra exhibit a small positive shift compared to that for Rhene, indicating the electron transfer from Rh metallene to Ir nanocluster.^[^ ^7^ ^]^ The X‐ray absorption spectroscopy (XAS) was further performed to scrutinize the local electronic and atomic structural information of Rh atoms. In comparison with Rh foil and Rhene, a slightly positive shift is discerned in the Rh K‐edge X‐ray absorption near‐edge structure (XANES) spectrum of Ir@Rhene (Figure 2b), further demonstrating the reduction of the electron density around the Rh sites after the incorporation of Ir element. As shown in the Fourier‐transformed extended X‐ray absorption fine structure (EXAFS) spectrum (Figure 2c; Table S1, Supporting Information), the prominent peak at about 2.4 Å can be attributed to Rh—Rh bonds.^[^ ^11^ ^]^ Moreover, a slight change in the coordination environment is observed, and the corresponding coordination number of Rh—Rh for Ir@Rhene is 9.2 ± 0.5, which is lower than that of 10.1 ± 0.5 for Rhene (Figure 2d). To further distinguish the Rh coordination environments, the wavelet transform spectra of these catalysts were conducted. As shown in Figure 2e, the spectra of both Rhene and Ir@Rhene exhibit the same feature area: a shell area for Rh—Rh scattering at *R* = 2.43 Å and *K* = 9.14 Å. Moreover, the Rh K‐edge *k* ^2^ *χ*(*k*) fitting curves of Ir@Rhene show a similar oscillation feature to that of the Rh foil and the Rhene and are markedly distinct from that of Rh_2_O_3_ (Figure S5, Supporting Information). This phenomenon reveals a similar atomic structure for the Rh site in both Ir@Rhene heterojunctions and Rh nanosheet, which is consistent with the wavelet transform spectra.

**Figure 3:**
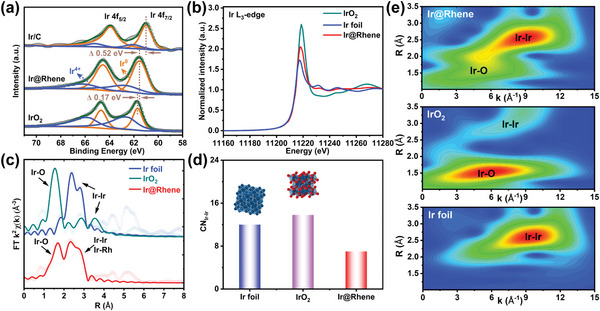
X‐ray absorption characterizations of Ir component in Ir@Rhenes heterostructure. a) Ir 4f XPS spectrum, b) XANES spectrum at the Ir L_3_‐edge, c) Fourier‐transformed (FT) of *k* ^2^‐weighted *χ*(*k*)‐function of the EXAFS spectrum, d) coordination numbers of various catalysts, and e) wavelet transform spectra of Ir@Rhene, IrO_2_, and Ir foil, respectively.

The Ir component in Ir@Rhene heterostructure was also investigated by analyzing the XPS and XAS spectra. **Figure** **3**a illustrates that the evident peak appeared at 61.0 eV could be assigned to Ir 4f_7/2_ of Ir/C, while the binding energy of Ir@Rhene shows an obvious positive shift about 0.52 eV. Moreover, the calculated content of Ir^4+^ for Ir@Rhene and IrO_2_ is 45.1 and 74.5 wt%, respectively, which is much higher than that of Ir/C (32 wt%), further indicating the formation of IrO_2_ species and electron transfer between Ir and O in the Ir@Rhene heterostructures. The XANES measurements were then carried out to gain more insight on the precise electronic information. As depicted in Figure 3b, the marked enhancement of the white‐line intensity of Ir@Rhene compared with Ir foil demonstrates a decreased occupation of the 5*d* state for Ir@Rhene and the formation of the high‐valent state Ir ions.^[^ ^12^ ^]^ Subsequently, the coordination environment of Ir atoms is analyzed by EXAFS (Figure S6, Supporting Information). Analogous to the Ir foil and IrO_2_ reference, the Ir@Rhene shows two major peaks at ≈1.7 and 2.5 Å, which could be derived from Ir—O and Ir—Ir bond, respectively (Figure 3c). Furthermore, the corresponding fitting results indicate that the coordination numbers of Ir—O and Ir—Rh for Ir@Rhene are 2.7 and 0.7, respectively (Table S2, Supporting Information). In parallel, the decreased coordination number of Ir—Ir in Ir@Rhene compared with Ir foil and IrO_2_ reference suggest the existence of substantial low‐coordinated Ir atoms (Figure 3d). The wavelet transform analysis was also carried out to shed more insight into the Ir species coordination information. The contour plot of Ir@Rhene clearly shows two intensities of the Ir—O (*k* ≈ 5.6 Å) and Ir—Ir (*k* ≈ 9.1 Å) coordination (Figure 3e), similar to the IrO_2_ (*k* ≈ 5.7 Å) and Ir foil (*k* ≈ 9.4 Å). These results demonstrate that the Ir atoms coexist as IrO_2_, metallic Ir, and IrRh alloy crystalline structures.

### 2.2 Electrocatalytic HER Performance

**Figure 4:**
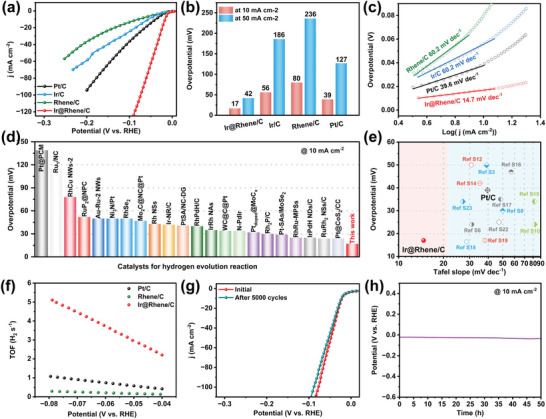
HER catalytic performances. a) Polarization curves of Ir@Rhene/C, Rhene/C, and commercial Pt/C and Ir/C. b) Comparison of the overpotentials at 10 and 50 mA cm^−2^. c) The corresponding Tafel plots. d) Overpotentials of Ir@Rhene/C at 10 mA cm^−2^ with recently reported PGMs‐based catalysts. e) HER performance comparison using Tafel slope versus overpotential (at 10 mA cm^−2^) for different PGMs‐based catalysts. f) Calculated H_2_ TOF comparison between Ir@Rhene and other catalysts. g) Polarization curves for the Ir@Rhene before and after 5000 cycles for stability test. h) 50 h chronopotentiometry curve of Ir@Rhene at 10 mA cm^−2^.

To evaluate the effects of unique geometric and electronic structures on catalytic performance, the HER activity of Ir@Rhene in alkaline media was conducted and compared alongside with that of the Rhene/C, commercial Pt/C, and Ir/C benchmark (Figure S8, Supporting Information). **Figure** **4**a shows that the Ir@Rhene/C presents the lowest overpotentials of 17 and 42 mV under the current densities of 10 and 50 mA cm^−2^, respectively, demonstrating excellent activity toward alkaline HER (Figure 4b). To elaborate on the potential HER kinetics behaviors, the corresponding Tafel plots were investigated and illustrated in Figure 4c. The resulting Ir@Rhene/C electrocatalysts deliver the fastest kinetics with the lowest slop of only 14.7 mV dec^−1^ among all the catalysts, indicating that Ir@Rhene undergoes a Volmer–Tafel pathway during the HER process.^[^ ^13^ ^]^ Moreover, the calculated exchange current density of Ir@Rhene was 1.01 mA cm^−2^, whereas those of Pt/C, Ir/C, and Rhene/C were 0.99, 0.98, and 0.94 mA cm^−2^, respectively, confirming that Ir@Rhene possesses superior intrinsic activity toward HER. Remarkably, Ir@Rhene delivers an ultrahigh mass activity (4.61 A mg_Ir+Rh_^−1^) at an overpotential of 50 mV, outperforming the commercial Pt/C (0.57 A mg_Ir+Rh_^−1^), the Ir/C (0.36 A mg_Ir+Rh_^−1^), and previously reported state‐of‐the‐art PGMs‐based HER catalysts, as summarized in Table S4 (Supporting Information). We next compare the overpotentials required to achieve 10 mA cm^−2^ and the Tafel slopes of Ir@Rhene heterostructure with many recently reported PGMs‐based HER catalysts, the Ir@Rhene delivers the highest electrocatalytic performance (Figure 4d,e; Table S5, Supporting Information).

To shed more light on the excellent electrocatalytic performance, we first compared the electrochemically active surface area (ECSA) of these electrocatalysts. Notably, the Ir@Rhene/C and Rhene/C exhibit a larger ECSA value than the commercial Pt/C and Ir/C due to their ultrathin properties, suggesting more electroactive sites exposed in Ir@Rhene/C (Figures S9 and S10, Supporting Information). In addition, the electrochemical impedance spectroscopy (EIS) results reveal that the Ir@Rhene/C has the smallest charge transfer resistance among the four catalysts, which implies a much faster charge transfer kinetics occurred at the interface of Ir@Rhene/C and the electrolyte (Figure S11, Supporting Information). Furthermore, Ir@Rhene/C presents a high turnover frequency (TOF) value of 2.21 H_2_ s^−1^ at an overpotential of 40 mV (Figure 4f), which is 5.3 and 17.0 times higher than that of Pt/C and Rhene/C, respectively, further indicating that the Ir@Rhene heterostructure has superior alkaline HER activity. Interestingly, we found that the yellow WO_3_ materials turn blue green with the aid of Ir@Rhene (Figure S12, Supporting Information), which clearly confirming the presence of the interfacial hydrogen spillover effect in the Ir@Rhene heterostructures.^[^ ^14^ ^]^ To further confirm this phenomenon, the cyclic voltammograms (CV) tests for Ir@Rhene/C, IrO_2_/C, IrRh/C, and Ir‐IrO_2_/C were carried out in H_2_‐saturated 1 m KOH solution. As shown in Figure S13 (Supporting Information), the minimum peak position shift caused by various scan rate in Ir@Rhene catalyst doubtlessly demonstrates the fastest hydrogen desorption kinetics,^[^ ^15^ ^]^ which boosting the interfacial synergistic catalysis effect between IrO_2_ and Ir/Rh sites. Besides activity, the Ir@Rhene demonstrates remarkable long‐term catalytic durability during the HER process. As depicted in Figure 4g, the polarization curve of Ir@Rhene loaded on the carbon paper does not exhibit an obvious negative shift after 5000 potential cycles. Moreover, the current–time chronopotentiometry measurement also indicates that Ir@Rhene possesses excellent electrochemical durability (Figure 4h), in which the overpotential of Ir@Rhene shows a negligible fluctuation after a 50‐h test. In addition, the morphology of Ir@Rhene/C is well maintained after the durability measurement (Figure S14, Supporting Information).

### 2.3 Reaction Mechanisms

**Figure 5:**
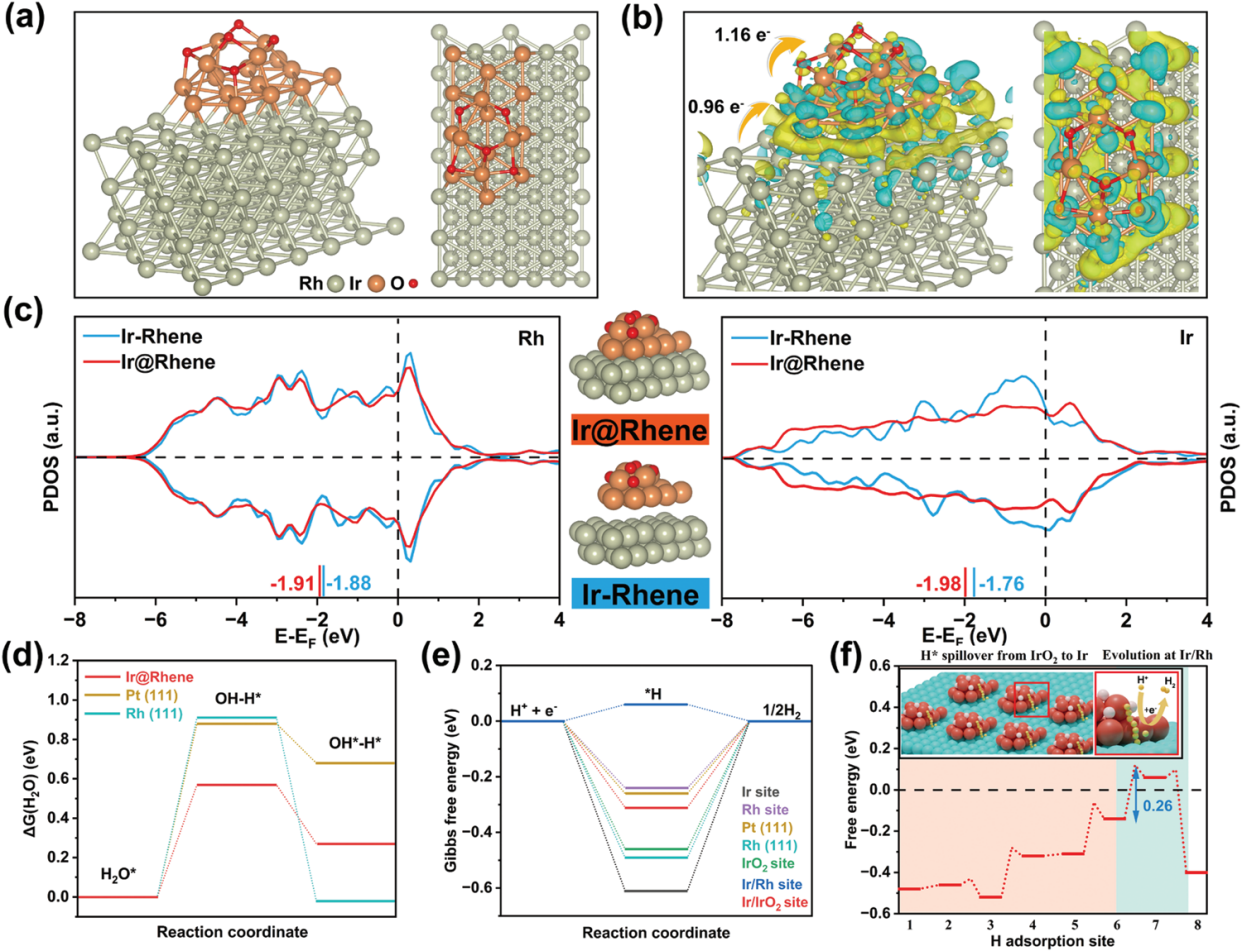
HER mechanisms and theoretical calculations for the Ir@Rhenes heterostructure. a) The theoretical model of Ir@Rhene. b) Differential charge density distribution and the electron transfer process. Yellow cloud indicates charge gain and cyan cloud represents charge depletion. c) Calculated PDOS plots of the Rh (left) and Ir (right) *d*‐band for Ir@Rhene and Ir‐Rhene, with aligned Fermi level. d) Comparison of water dissociation energy barrier for Ir@Rhene, Pt (111), and Rh (111). e) The Δ*G*_H*_ of various sites and models. f) Reaction free energy diagram for hydrogen spillover on various catalytic sites along the IrO_2_ region to the Ir/Rh interface of the Ir@Rhene. (Inset: Schematic diagram of hydrogen spillover effect.)

With these experimental results in hand, we now turn to employ theoretically the density functional theory (DFT) to scrutinize mechanisms responsible for the superiority of Ir@Rhene in alkaline HER. Based on the results of X‐ray absorption characterizations, the Ir@Rhene heterostructural model was established by placing an Ir/IrO_2_ Janus nanoparticle on the ultrathin bulk substrate of the (2 × 3) supercells of the Rh metallene (**Figure** **5**a). To fully investigate the electronic structure–intrinsic activity underpinnings of the interfacial effects, the details regarding the Bader charge and the differential charge density are first analyzed. As shown in Figure 5b, the formation of the heterostructured interface allows the electron transfer from Rh to Ir and ultimately to O atoms, which facilitates water adsorption around the O atoms in the IrO_2_ site.^[^ ^16^ ^]^ In addition, the projected density of states (PDOS) of the Ir@Rhene heterostructure was further investigated and compared alongside with the physically connected Ir nanoclusters and Rh (111) structure (denoted as Ir‐Rhene, Figures S15 and S16, Supporting Information). From the negative shift of PDOS as illustrated in Figure 5c, the center of the *d*‐band for both the Rh and Ir atoms are far away from the Fermi level, implying the decrease in the chemisorption energy of H intermediate on the Ir@Rhene surface and thus improve the HER kinetics.^[^ ^17^ ^]^ Since the efficiency of the alkaline HER relies on both the water dissociation energy barrier and the H* adsorption/desorption behaviors on the catalyst surface,^[^ ^18^ ^]^ thus the water adsorption energies (*E*_water_) on various sites were first calculated to identify the most favorable adsorption site for water dissociation (Figure S17, Supporting Information). Significantly, the strongest binding behavior of H_2_O* is observed on the IrO_2_ site (—0.60 eV), surpassing that of the Ir site (—0.50 eV), the Rh site (—0.53 eV), and the Ir/Rh interface site (—0.51 eV). Meanwhile, the energy barrier for water dissociation on this particular site is only 0.57 eV (Figure 5d and Figures S18–S20, Supporting Information), which is considerably lower than that of Pt (0.88 eV), Rh (0.91 eV), and other sites of Ir@Rhene (Figure S21, Supporting Information). Furthermore, IrO_2_ site shows weaker adsorption behavior of OH*, avoiding the poisoning of the catalytic site and eventually making it has a better catalytic performance (Figure S22, Supporting Information).^[^ ^19^ ^]^ These findings suggest that water molecules are preferably adsorbed at the IrO_2_ site of Ir@Rhene with significantly lower dissociation energy, implying that this heterostructure can facilitate water adsorption and dissociation into desired H* intermediates. We further calculated the Gibbs free energies of adsorbed atomic hydrogen (Δ*G*_H*_) on Ir@Rhene with different sites to explore hydrogen adsorption/desorption behaviors and corresponding atomic configurations are shown in Figure S23 (Supporting Information). Figure 5e indicates that Rh and Pt have a high Δ*G*_H*_ of —0.49 and —0.26 eV, respectively, which suggests that hydrogen desorption is their potential‐determining step.^[^ ^20^ ^]^ In contrast, the Ir/Rh interface site shows the most appealing upslope of 0.06 eV, compared to those of Rh (—0.24 eV), Ir (—0.61 eV), IrO_2_ (—0.46 eV), and Ir/IrO_2_ interface (—0.31 eV) sites, suggesting more greatly balanced adsorption/desorption behaviors around this site than the others.^[^ ^21^ ^]^ These results imply that the water dissociation and combination of H* generating H_2_ in our catalyst model occur at different sites. Therefore, the work function difference (ΔΦ) between each component of Ir@Rhene was first determined to evaluate whether the interfacial hydrogen spillover is present or not. As shown in Figure S24 (Supporting Information), the small ΔΦ between IrO_2_ and Ir and between Ir and Rh in three‐phase heterostructure (IrO_2_/Ir/Rh) could induce interfacial charge dilution and relocation and weaken the interfacial H* trapping,^[^ ^15^, ^22^ ^]^ thereby supplying a hydrogen spillover path of IrO_2_ → Ir → Ir/Rh. With these work functions enabled, the energy barrier for hydrogen evolution will be significantly reduced, and was further demonstrated by Figure 5f and Figure S25 (Supporting Information). As expected, the energy barrier of hydrogen evolution is only 0.26 eV (sites 6–7) after hydrogen spillover process. Overall, our results demonstrate that the hydrogen atoms dissociated from the IrO_2_ site rapidly occupy the nearby region, and then migrate to the Ir/Rh interface site through interfacial hydrogen spillover, ultimately achieving hydrogen desorption. Therefore, during the whole water splitting process, the remarkable cooperation between the IrO_2_ and Ir/Rh interface site enables them to prevent steric blocking of the active site and improve HER kinetics on the Ir@Rhene heterostructures.

## 3 Conclusion

In summary, new Ir@Rhene heterostructures were successfully obtained via confining sub‐nm Ir/IrO_2_ Janus nanoclusters on the ultrathin Rh metallene toward accelerated hydrogen evolution kinetics in alkaline media for efficient water splitting. The unique cooperation between IrO_2_ and Ir/Rh interface triggers and improves the interfacial hydrogen spillover effect, accelerating the whole alkaline HER kinetics, with an ultralow Tafel slope of 14.7 mV dec^−1^ and an overpotential of only 17 mV at 10 10 mA cm^−2^. Our work demonstrates the feasibility of integrating all favorable features into one catalyst system by the heterointerface engineering strategy, and opens up new opportunities for designing and constructing advanced catalysts to be used for broad thermal and electrochemical catalytic reactions.

## 4 Experimental Section

### Materials

Rhodium (III) acetylacetonate (Rh(acac)_3_, 97%) was received from Sigma‐Aldrich. Poly(vinylpyrrolidone) (PVP, K30), formaldehyde aqueous solution (37.0%–40.0%), and benzyl alcohol were purchased from Sinopharm chemical reagent Co., Ltd. Potassium hydroxide (KOH, 99.99%), iridium chloride hydrate (IrCl_3_·*x*H_2_O, 99.9%), ethylene glycol (EG, 99%), and ethanol were obtained from Aladdin. Nafion solution and commercial Pt/C were acquired from Alfa Aesar. Commercial Ir/C was purchased from Premetek Company.

### Synthesis of Rhene

In a standard preparation of Rhene, 8 mg of Rh(acac)_3_, 120 mg of PVP, 3 mL of formaldehyde solution, and 3 mL of benzyl alcohol were first added into a 25 mL Teflon‐lined autoclave. After stirring at 950 rpm for 1 h, the homogenous mixture was rapidly heated to 180 °C in 30 min and maintained at 180 °C for 8 h. After naturally cooling to room temperature, the Rhene as black products were collected by centrifugation at 12 000 rpm for 15 min, followed by washing twice with an acetone/ethanol mixture and once with ethanol. Finally, the Rhene was dried for further use.^[^ ^9^ ^]^

### Synthesis of Ir@Rhene Heterostructures

In a typical synthesis, 4 mg of as‐prepared Rhene was dispersed in 30 mL of EG‐water (v/v = 1:1) solution under ultrasonication. After around 30 min, 5.3 mg of IrCl_3_·xH_2_O was added into the resulting solution and sonicated for another 20 min. The homogeneous suspension was then heated at 185 °C in the 50 mL Teflon‐lined autoclave for 3 h. The resulting products were separated through centrifugation at 12 000 rpm for 10 min, followed by washing with ethanol three times. Finally, the products were dried via Vacufuge at 45 °C for further use.

### Structural and Compositional Analyses

The morphological and structural analysis was determined by TEM (JEOL, JEM‐2100plus, 200 kV). The aberration‐corrected HRTEM, HADDF‐STEM, and EDS mapping were recorded on Titan Cubed Themis G2300. The SEM and corresponding EDS mapping were conducted on ZEISS Gemini 300. The XRD patterns were collected on a Rigaku D/max‐2200 PC diffractometer with Cu K*α* radiation (*λ* = 1.5418 Å). The XPS data of different catalysts were tested using a Thermo ESCALAB Xi+ instrument equipped with an Al‐K*α* radiation source (1486.6 eV). The thickness of different catalysts was determined by AFM (Bruker, Multimode 8). The XAS of the samples were collected at the beamline BL14W1 of the Shanghai Synchrotron Radiation Facility (SSRF, China). The ICP‐MS was conducted on a PerkinElmer NexION 350D.

### Electrochemical Measurements

All electrochemical evaluations were carried out using a CS2350 electrochemical workstation (Wuhan Corrtest Instrument Co., Ltd., China) with a standard three‐electrode system at room temperature. Particularly, an L‐type glassy carbon electrode (GCE, diameter: 3 mm, area: 0.071 cm^2^) coated with various electrocatalysts acts as the working electrode. The graphite rod and the Hg/HgO electrode were utilized as the counter electrode and the reference electrode, respectively. The surface of GCE was first polished with Al_2_O_3_ slurry before electrochemical testing and then cleaned with Milli‐Q water and ethanol. To fabricate the working electrode, taking Ir@Rhene catalyst ink solution as an example, 4 mg Vulcan XC‐72 substrates were thoroughly dispersed with 1 mg catalysts in 1.25 mL mixed solution containing 475 µL of deionized water, 475 µL of ethanol, and 50 µL of Nafion solution under ultrasonic treatment. Then, 2 µL of the as‐prepared catalyst ink (4 mg mL^−1^) was pipetted onto the clean GCE surface and dried naturally. The final loading concentration for the different electrocatalysts on the working electrode was determined using ICP‐MS.

The Hg/HgO electrode was also calibrated in the highly pure H_2_‐saturated 1 m KOH electrolyte using a Pt plate as the working electrode (Figure S7, Supporting Information), the measured potentials with respect to the reversible hydrogen electrode (RHE) in the experiments were converted as follows: *E*(RHE) = *E*(Hg/HgO) + 0.914 V. All polarization curves were carried out in an N_2_‐saturated 1 m KOH electrolyte with a sweep rate of 5.0 mV s^−1^ and corrected with 95% *IR* compensation. The stability test for the Ir@Rhene electrocatalyst loaded on the carbon paper with the loading of 0.2 mg_metal_ cm^−2^ was conducted using chronopotentiometry in 1.0 m KOH solution at a continuous current density of 10 mA cm^−2^. The electrochemical active surface areas (ECSA) were calculated from the H desorption region for CV curves in 1 m KOH solution by using a charge density of 210 µC cm^−2^ for Pt/C, 218 µC cm^−2^ for Ir/C, 221 µC cm^−2^ for Rhene/C and Ir@Rhene/C.^[^ ^23^ ^]^

### Theoretical Calculation Methods

To scrutinize the effects of the unique interfacial structure of Ir@Rhene heterojunctions toward alkaline HER, the slabs of Ir@Rhene, Ir‐Rhene, Pt (111), and Rh (111) were established, respectively. The Ir@Rhene was modeled by using a 2 × 3 × 1 supercell. All density functional theory (DFT) calculations of these slabs were performed using the Perdew–Burke–Ernzerhof (PBE) exchange‐correlation functional with the kinetics energy cutoff of 400 eV at the Vienna Ab initio Simulation Package (VASP, version 5.4.1).^[^ ^24^ ^]^ The projected augmented wave (PAW) method^[^ ^25^ ^]^ and empirical DFT‐D3 correction method^[^ ^26^ ^]^ were used to describe the ion–electron interactions and the weak interaction, respectively. The convergence criteria for geometry optimizations and electron energies were set as 0.02 eV Å^−1^ and 10^−4^ eV, respectively. A vacuum layer of 15 Å was incorporated onto the surface of slabs to avoid artificial interactions. H_2_O and H_2_ were calculated in boxes of 15 Å × 15 Å × 15 Å, with the gamma point only.

## Conflict of Interest

The authors declare no conflict of interest.

## Supporting information

Supporting Information

## Data Availability

The data that support the findings of this study are available from the corresponding author upon reasonable request.
