# Supplementary material for: Modulating Water Splitting Kinetics via Charge Transfer and Interfacial Hydrogen Spillover Effect for Robust Hydrogen Evolution Catalysis in Alkaline Media
Source: Adv Sci (Weinh). 2023 Jun 23;10(24):2302358. doi: 10.1002/advs.202302358 (PMC10460870; doi:10.1002/advs.202302358)
Supplement: Supplementary file 1 — Supporting Information [file ADVS-10-2302358-s001.pdf]

ADVANCED SCIENCE
Open Access

Supporting Information

for *Adv. Sci.*, DOI 10.1002/advs.202302358

Modulating Water Splitting Kinetics via Charge Transfer and Interfacial Hydrogen Spillover Effect for Robust Hydrogen Evolution Catalysis in Alkaline Media

*Yiming Jiang, Juncai Leng, Shiqi Zhang, Tingyi Zhou, Mingxuan Liu, Shuoming Liu, Yahui Gao, Jianwei Zhao, Lei Yang, Li Li and Wei Zhao**

Supporting Information

Modulating Water Splitting Kinetics via Charge Transfer and Interfacial Hydrogen Spillover Effect for Robust Hydrogen Evolution Catalysis in Alkaline Media

*Yiming Jiang, Juncai Leng, Shiqi Zhang, Tingyi Zhou, Mingxuan Liu, Shuoming Liu, Yahui Gao, Jianwei Zhao, Lei Yang, Li Li, Wei Zhao**

Dr. Y. Jiang, J. Leng, S. Zhang, T. Zhou, M. Liu, S. Liu, Y. Gao, Prof. L. Li, Prof. W. Zhao

State Key Laboratory of Food Science and Technology, School of Food Science and Technology, Jiangnan University, Wuxi, Jiangsu 214122, P. R. China

*Corresponding author, Email: zhaow@jiangnan.edu.cn

J. Zhao, L. Yang

Shenzhen HUASUAN Technology Co. Ltd., Shenzhen 518055, P. R. China

Supplementary Results

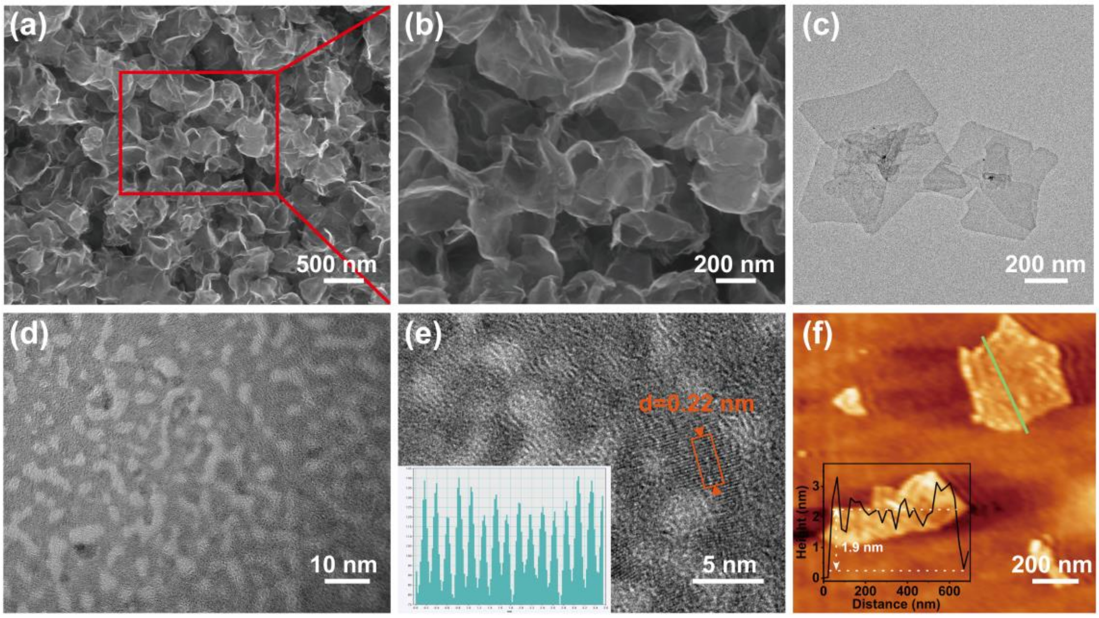

Figure S1. Characterization of Rhenes. (**a**, **b**) SEM images, (**c**) low-magnification TEM image, (**d**) TEM image of Rhene after beam exposure for 5 s and (**e**) corresponding high-resolution TEM image, and (**f**) AFM image and corresponding height profile.

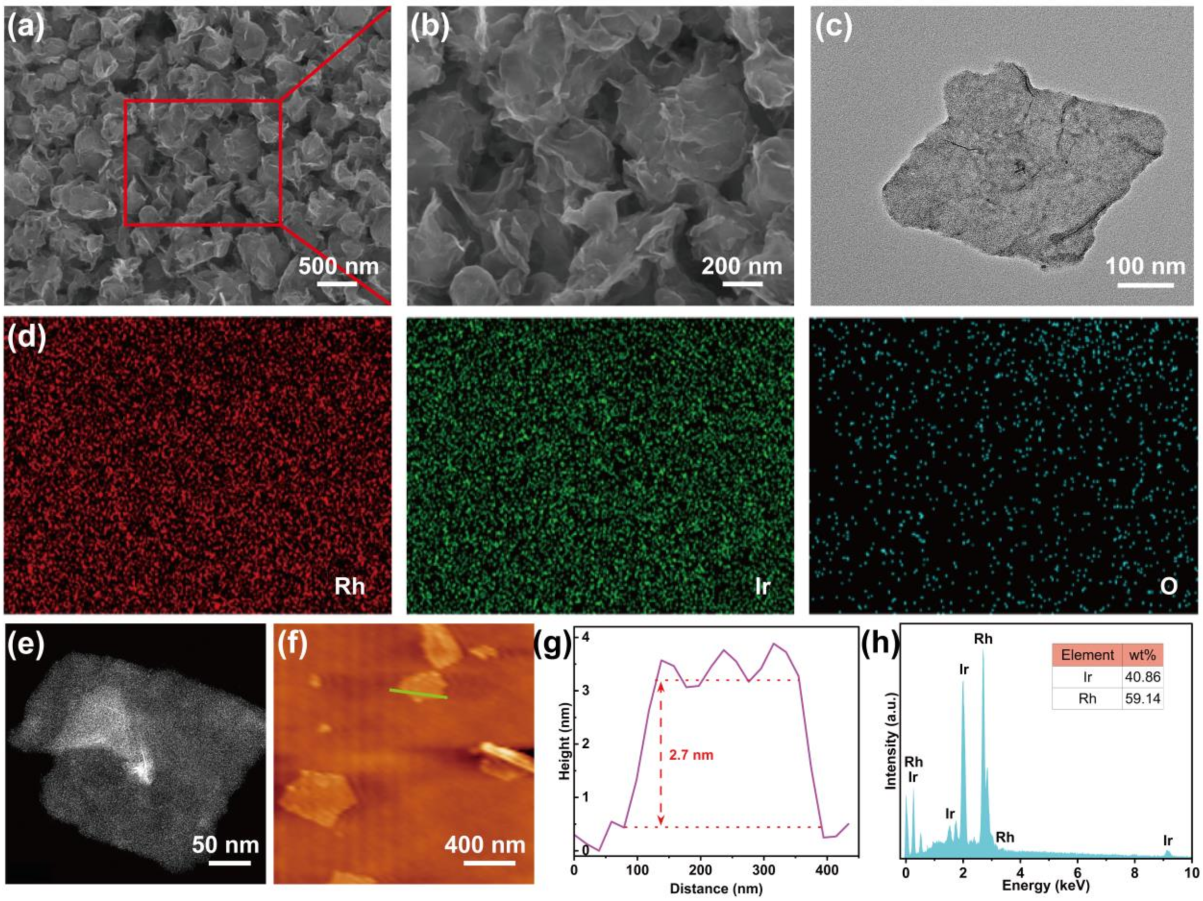

Figure S2. Characterization of Ir@Rhenes. (**a**, **b**) SEM images and (**d**) corresponding EDS mapping images, (**c**) low-magnification TEM image, (**e**) HADDF-STEM image, (**f**) AFM image and (**g**) corresponding height profile, and (**h**) atomic composition determined by SEM-EDS.

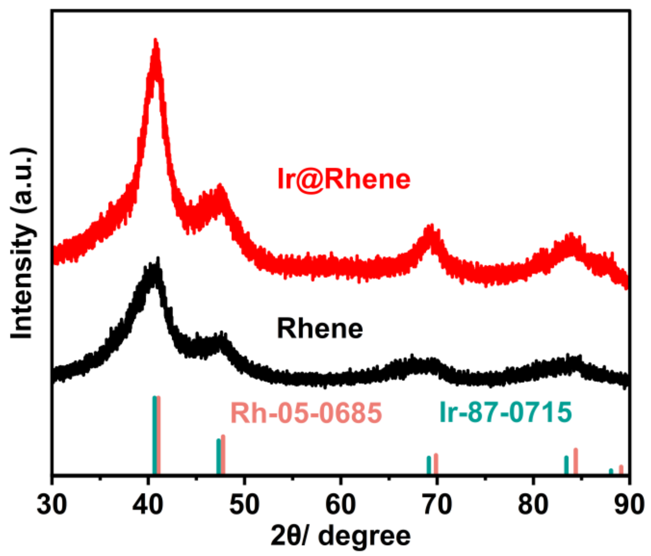

Figure S3. XRD patterns of Ir@Rhene and Rhene.

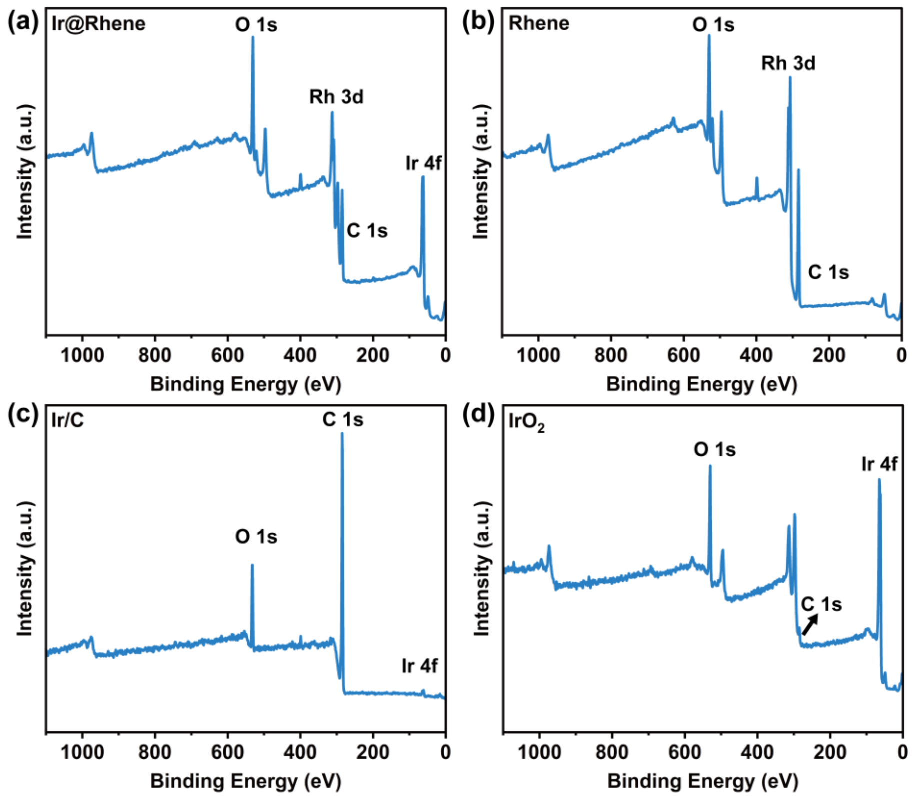

Figure S4. XPS survey spectra of (**a**) Ir@Rhene, (**b**) Rhene, (**c**) Ir/C, and (**d**) IrO_2.

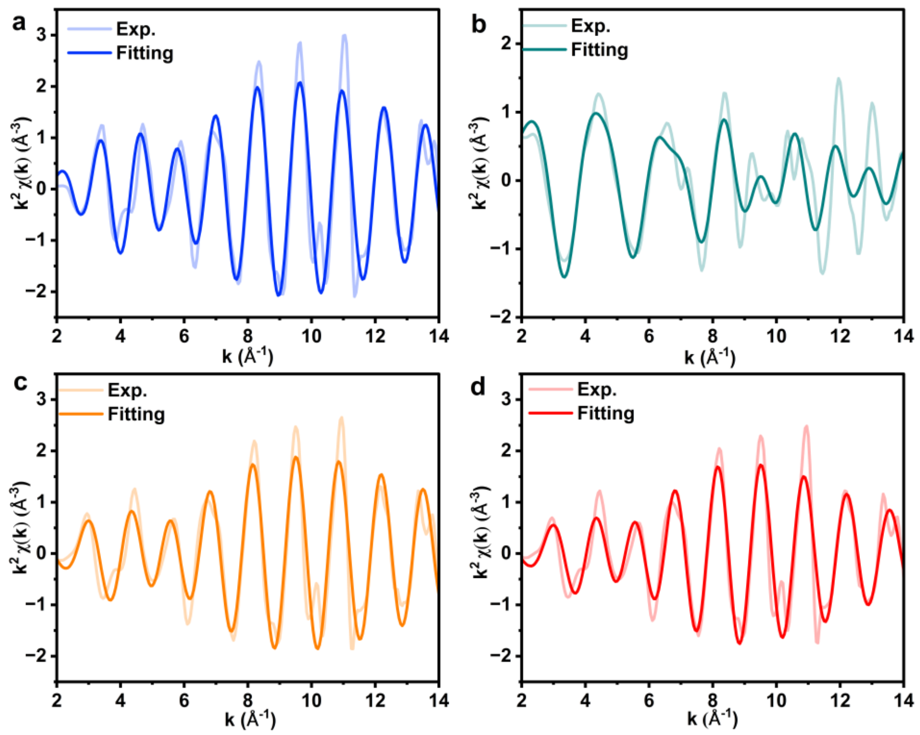

Figure S5. The Rh K-edge $k^2\chi(k)$ oscillations curves of Rh foil, Rh_2O_3, Rhene, and Ir@Rhene, respectively.

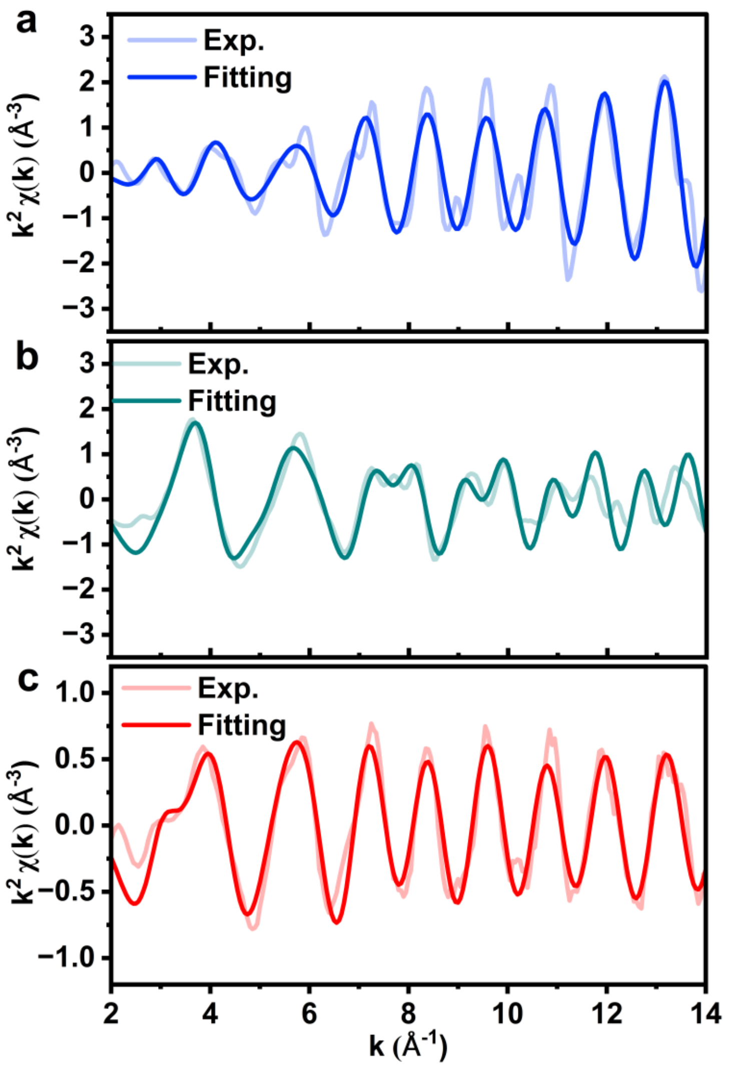

Figure S6. The Ir L_3-edge $k^2\chi(k)$ oscillations curves of Ir foil, IrO_2, and Ir@Rhene, respectively.

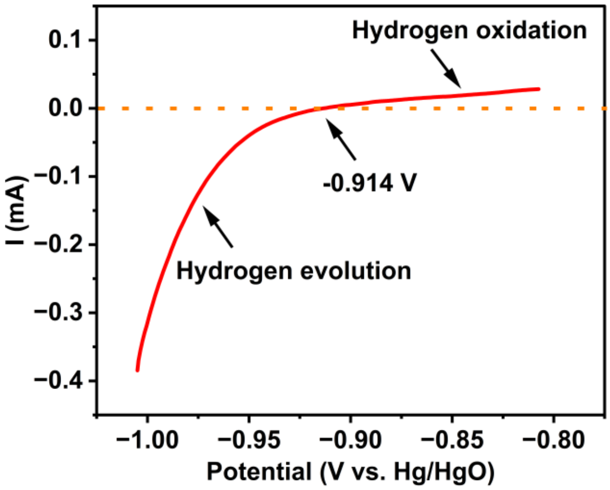

Figure S7. Current–potential curve of Pt plate in highly pure H_2-saturated 1.0 M KOH electrolyte, used for calibration of the Hg/HgO electrode with respect to RHE. Scan rate: 1 mV s^{-1}.

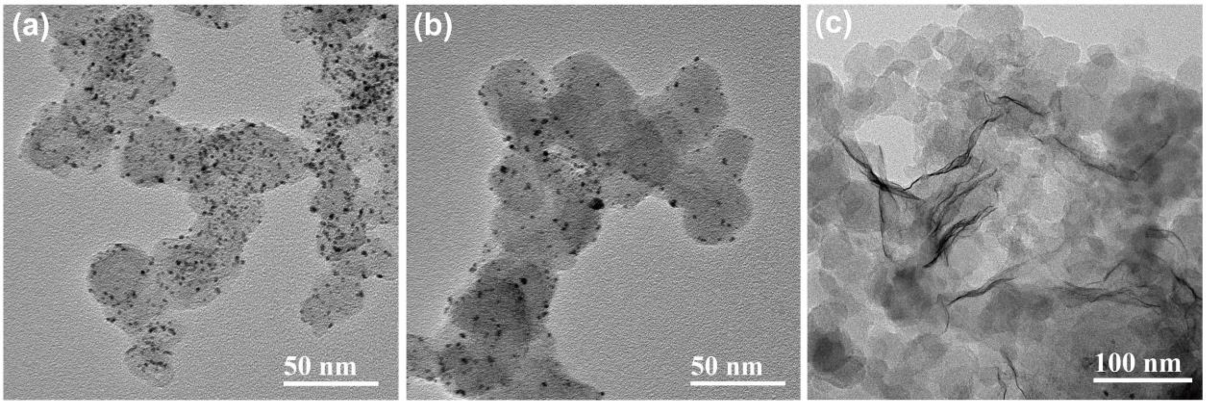

Figure S8. TEM images of (**a**) Pt/C, (**b**) Ir/C, and (**c**) Rhene/C.

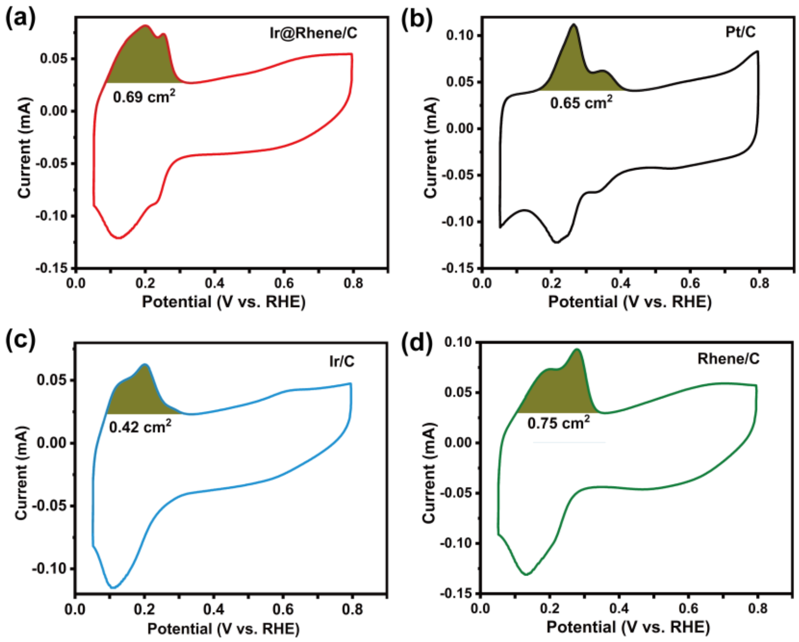

Figure S9. CV curves of (**a**) Ir@Rhene/C, (**b**) Pt/C, (**c**) Ir/C, and (**d**) Rhene/C in N_2-saturated 1 M KOH solution with a scan rate of 50 mV s^{-1}.

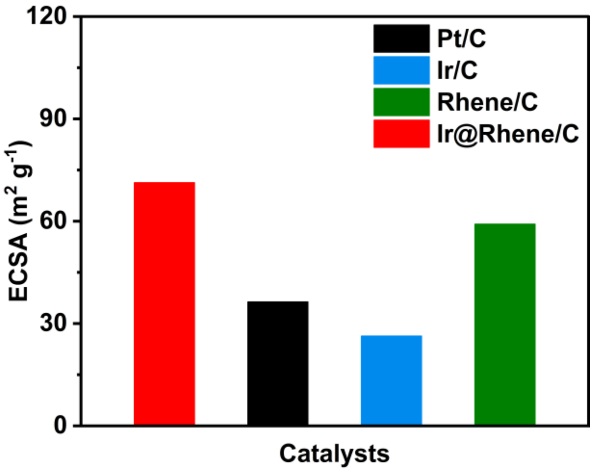

Figure S10. The ECSA of Ir@Rhene/C, Pt/C, Ir/C, and Rhene/C.

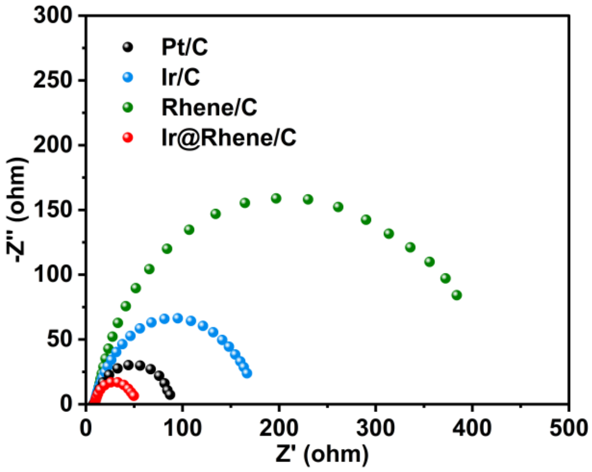

Figure S11. Nyquist plots of Ir@Rhene/C, Rhene/C, and commercial Pt/C and Ir/C catalysts at an overpotential of 50 mV (*vs*. RHE), respectively.

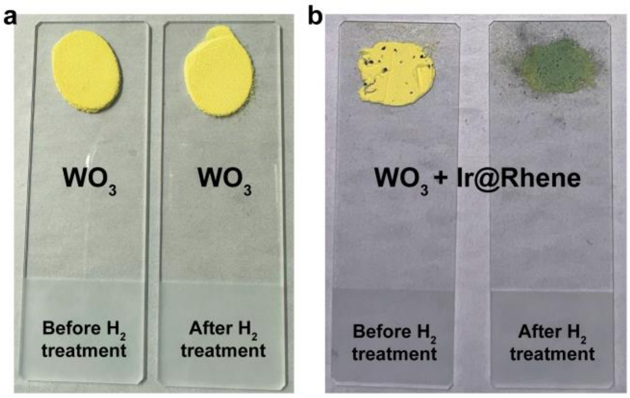

Figure S12. The determination of interfacial hydrogen spillover for Ir@Rhene. a) the photographic image of WO_3 before and after H_2 treatment at room temperature (25 ^{o}C), observing that the WO_3 does not undergo an observable color change. **b)** the photographic image of physically mixed materials for WO_3 and Ir@Rhene before and after H_2 treatment at room temperature (25 ^{o}C), clearly showing the color change from yellow to blue green. (**Caution**: the flow rate of H_2 gas must be controlled at lower flow velocity due to the produced spark and the risk of hydrogen gas burning).

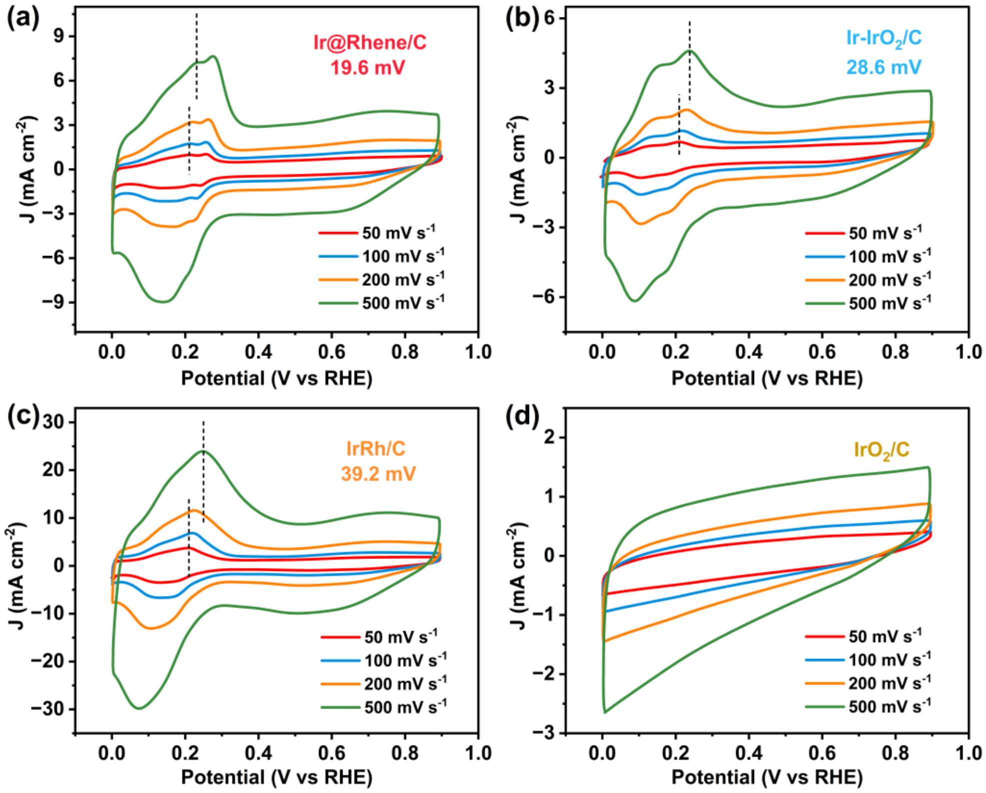

Figure S13. CV curves for (**a**) Ir@Rhene/C, (**b**) Ir-IrO_2/C, (**c**) IrRh/C, and (**d**) IrO_2/C. These CV curves were performed at various scan rates (50, 100, 200, and 500 mV s^{-1}) in H_2-saturated 1 M KOH solution.

For the synthesis of IrRh/C, 6 mg Vulcan XC-72 substrates, 4.0 mg $IrCl_3 \cdot xH_2O$, and 6.3 mg $Na_3RhCl_6 \cdot 12H_2O$ dispersed in 30 mL EG solution under ultrasonication. The homogeneous suspension was then heated at 185°C in the 50 mL Teflon-lined autoclave for 3h. the resulting products were centrifugated and washed by ethanol three times, followed by dried via vacufuge at 45°C.

For the IrO_2/C, 2.6 mg IrO_2 and 10.4 mg XC-72 substrates were thoroughly dispersed in a mixture solution containing 1.543 mL of ethanol, 1.543 mL of DI water, and 0.163 mL Nafion solution to prepare catalyst ink (4 mg mL^{-1}).

For the Ir-IrO_2/C, 12 mg Ir/C and 2.4 mg IrO_2 were thoroughly dispersed in a mixture solution containing 1.71 mL of ethanol, 1.71 mL of DI water, and 0.18 mL of Nafion solution to prepare catalyst ink (4 mg mL^{-1}).

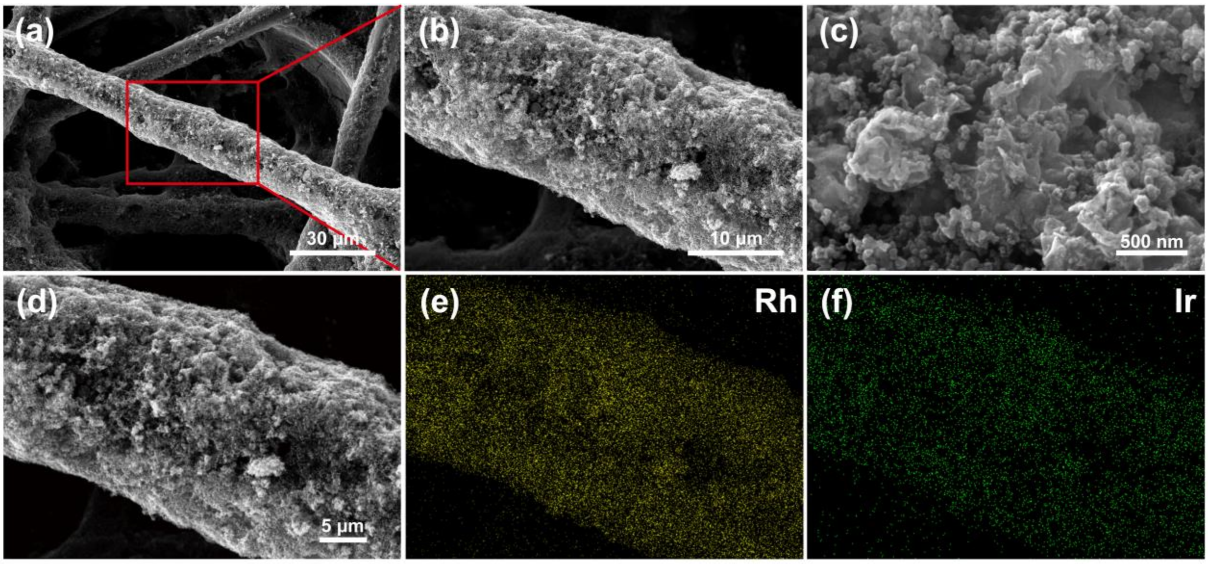

Figure S14. SEM images and corresponding EDS-mapping images of the Ir@Rhene/C on carbon paper after durability measurement.

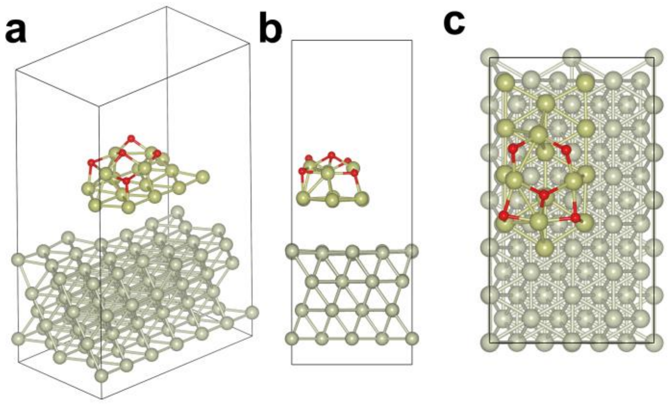

Figure S15. Theoretical model of Ir-Rhene, showing the **a**) oblique view, **b**) front view, and **c**) top view of model.

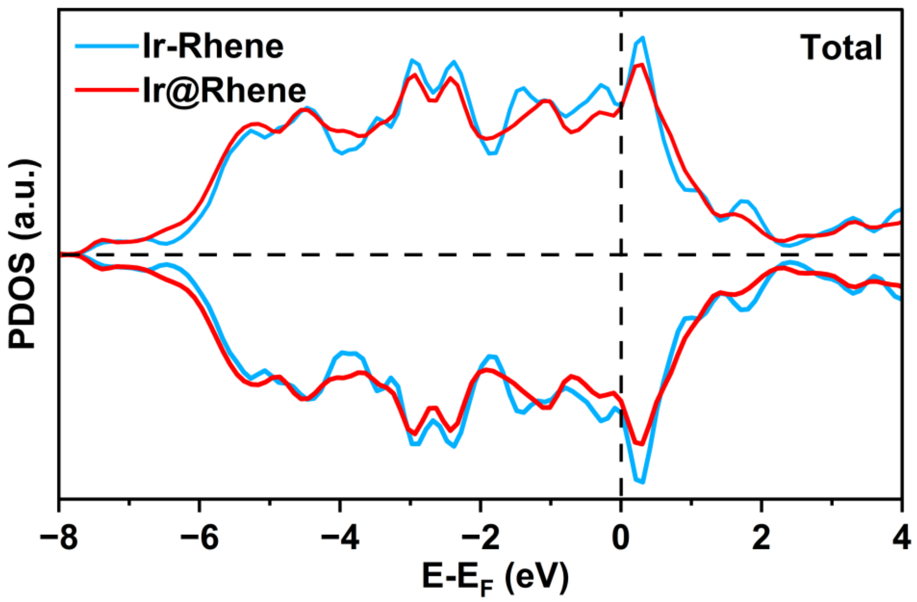

Figure S16. Total DOS plots of Ir@Rhene and Ir-Rhene.

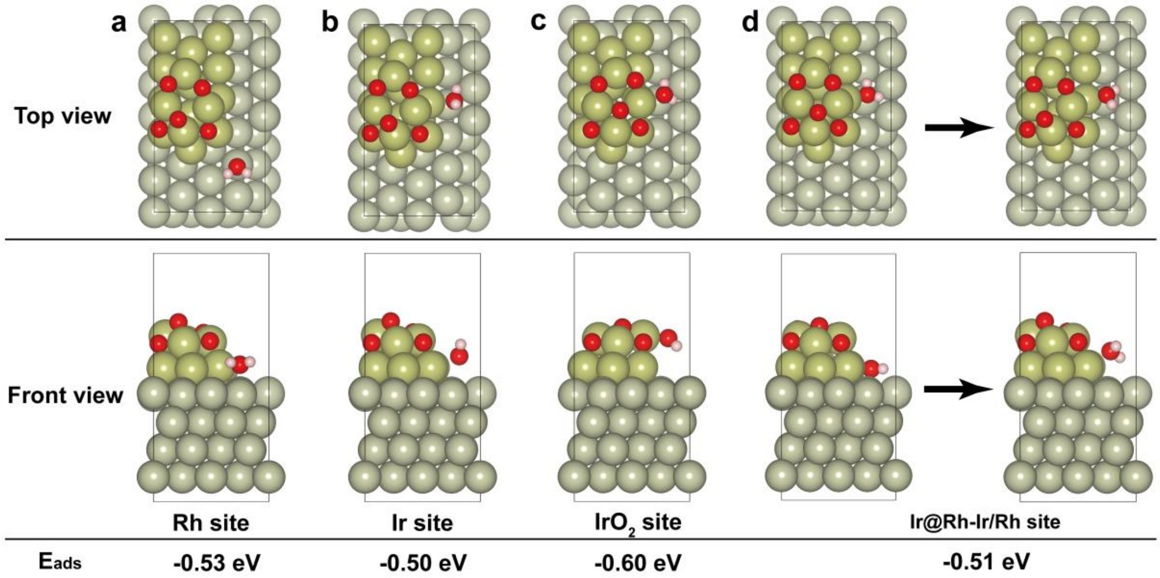

Figure S17. The optimized configurations of water adsorption (H_2O*) on the (**a**) Rh site, (**b**) Ir site, (**c**) IrO_2 site, and (d) Ir/Rh interface site of Ir@Rhene model, showing both the top view and front view of each structure, as well as the corresponding adsorption energy (E_{ads}).

The Figure S17 shows adsorption configurations of water molecule at various sites of Ir@Rhene model. Interestingly, the water molecule placed on the Ir/Rh interface site is unstable and slips to the adjacent Ir site spontaneously during the geometry optimization. These findings further demonstrate that the H_2O adsorption and dissociation are more favorable at the IrO_2 site.

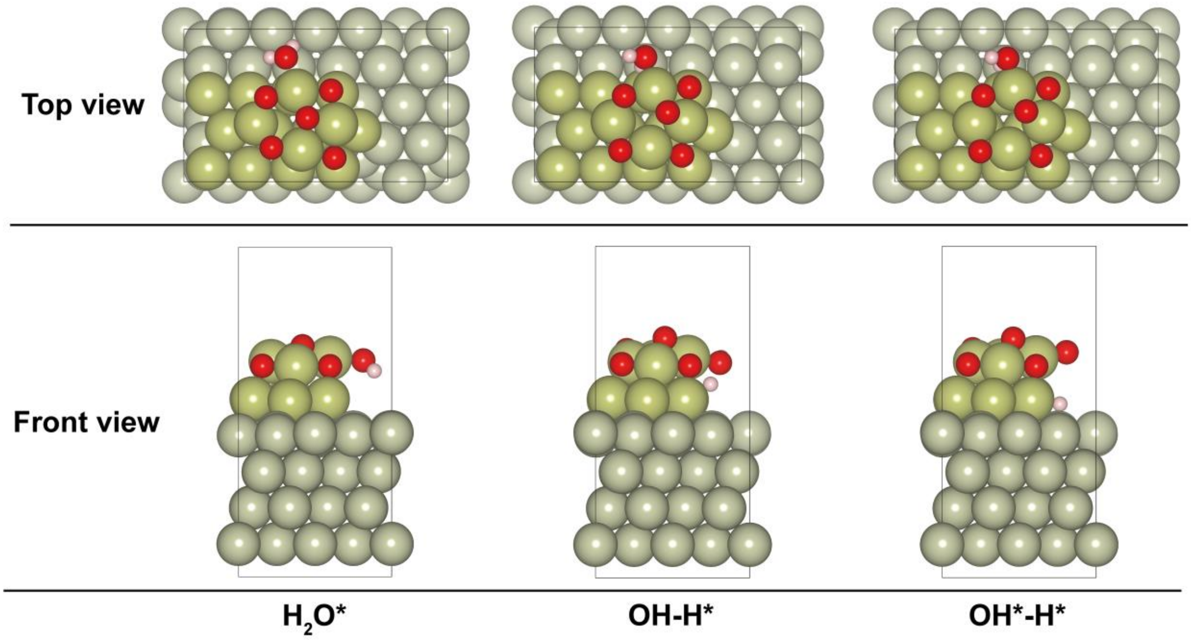

Figure S18. Atomic configurations of Ir@Rhene during the H_2O dissociation into OH* and H*.

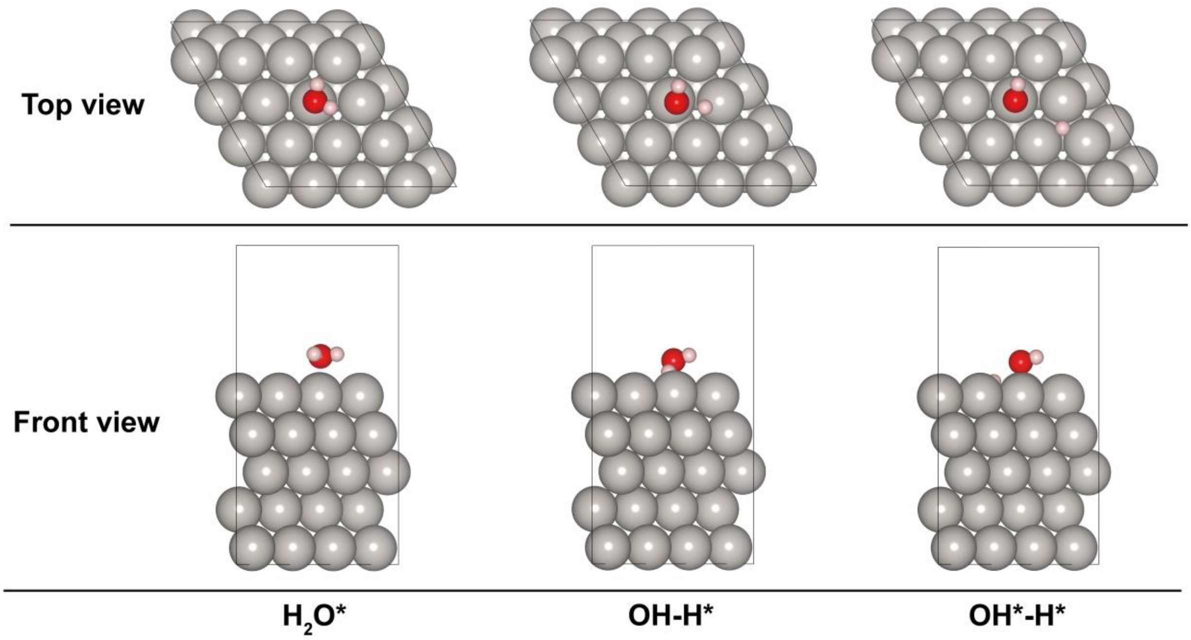

Figure S19. Atomic configurations of Pt (111) slab during the H_2O dissociation into OH* and H*.

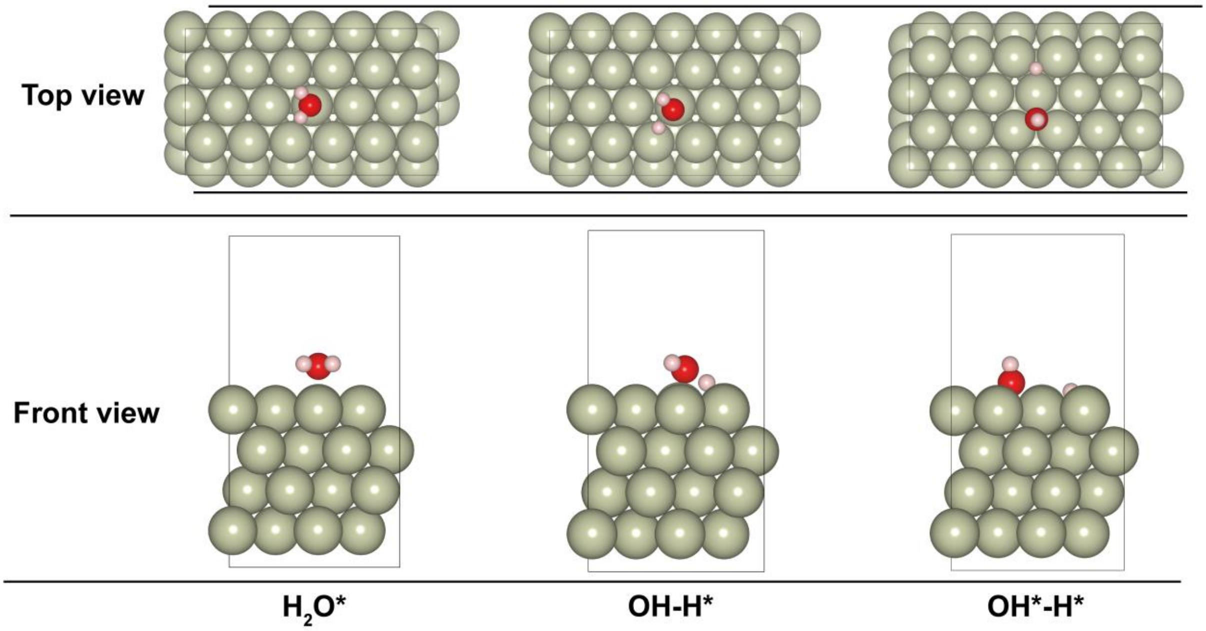

Figure S20. Atomic configurations of Rh (111) slab during the H_2O dissociation into OH* and H*.

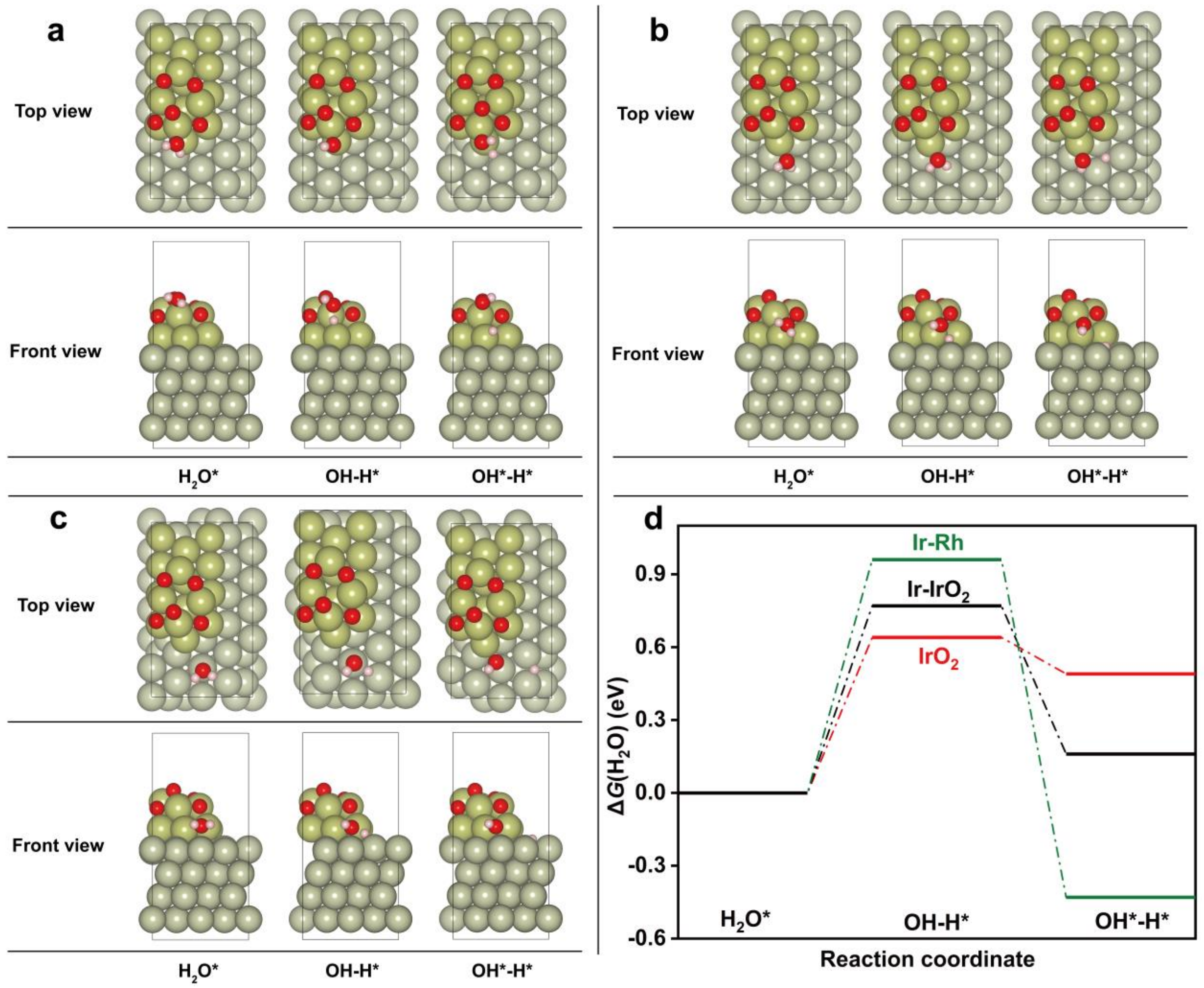

Figure S21. Atomic configurations of (**a**) IrO_2 site, (**b**) Ir-IrO_2 site, and (**c**) Ir-Rh site of Ir@Rhene during the H_2O dissociation into OH* and H*, and (**d**) corresponding water dissociation energy barrier.

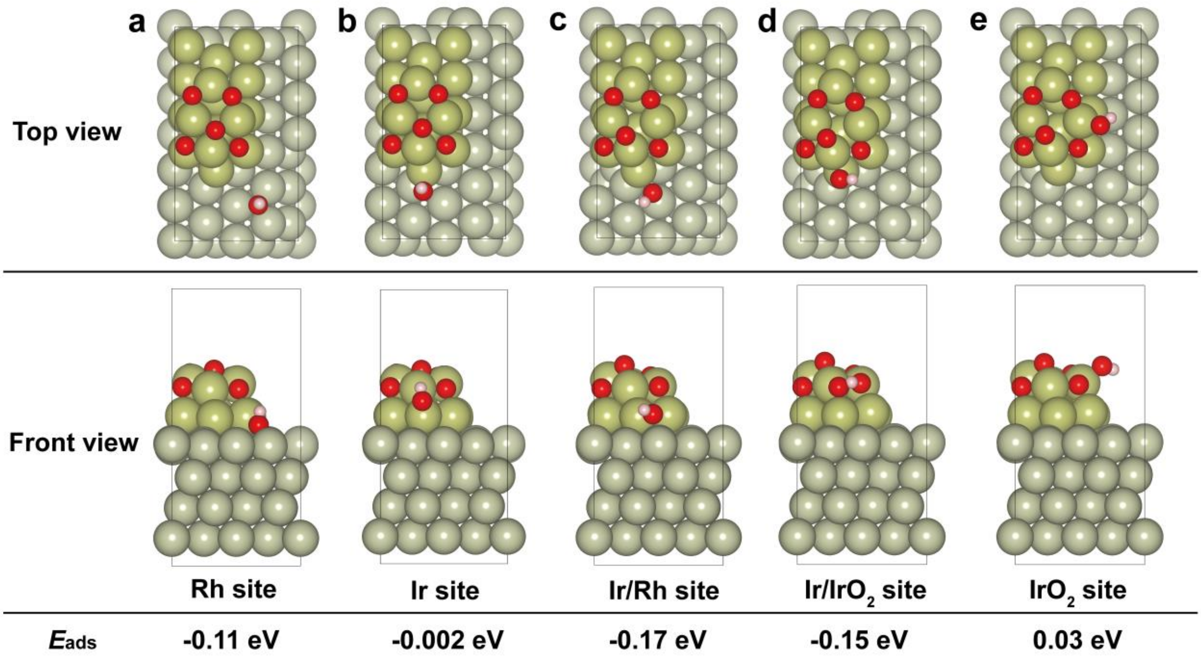

Figure S22. The optimized configurations of hydroxyl adsorption (OH*) on the (**a**) Rh site, (**b**) Ir site, (**c**) Ir/Rh site, (**d**) Ir/IrO_2 site, and (**e**) IrO_2 site of Ir@Rhene model, showing both the top view and front view of each structure, as well as the corresponding adsorption energy (E_{ads}).

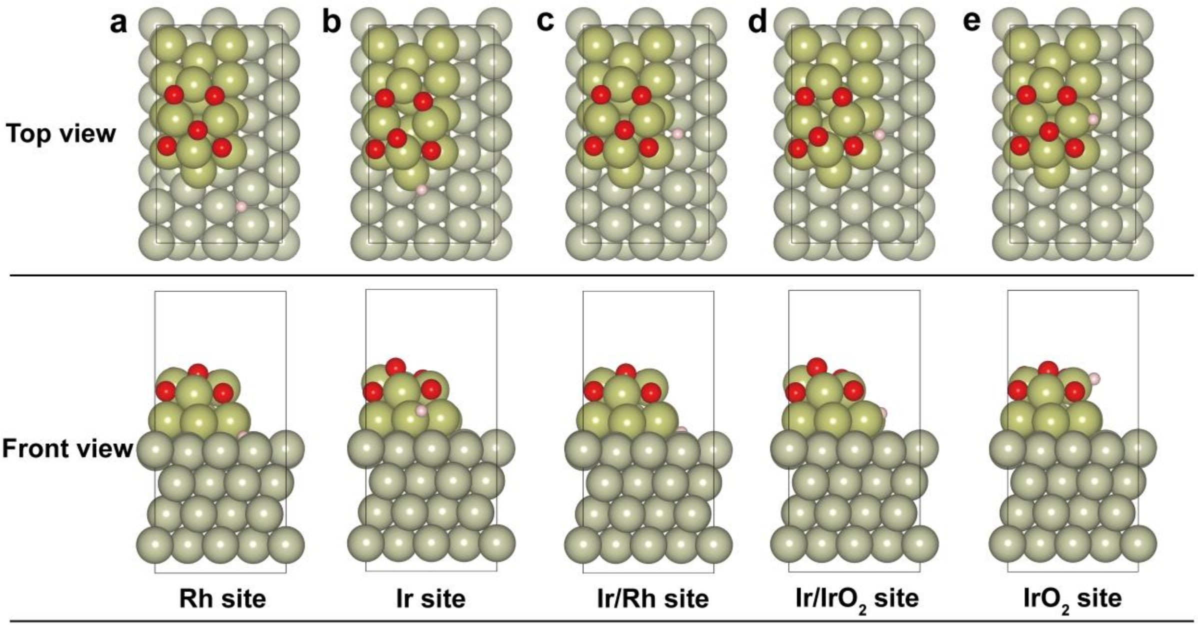

Figure S23. The optimized configurations of hydrogen adsorption (H*) on the (**a**) Rh site, (**b**) Ir site, (**c**) Ir/Rh interface site, (**d**) Ir/IrO_2 interface site, and (**e**) IrO_2 site of Ir@Rhene model, showing both the top view and front view of each structure.

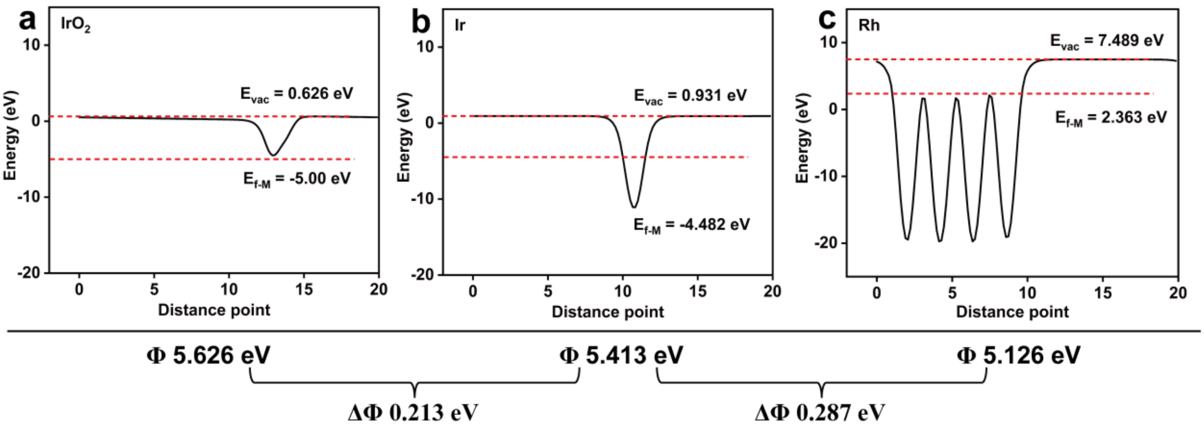

Figure S24. Work function of (**a**) IrO_2, (**b**) Ir, and (**c**) Rh of Ir@Rhene model.

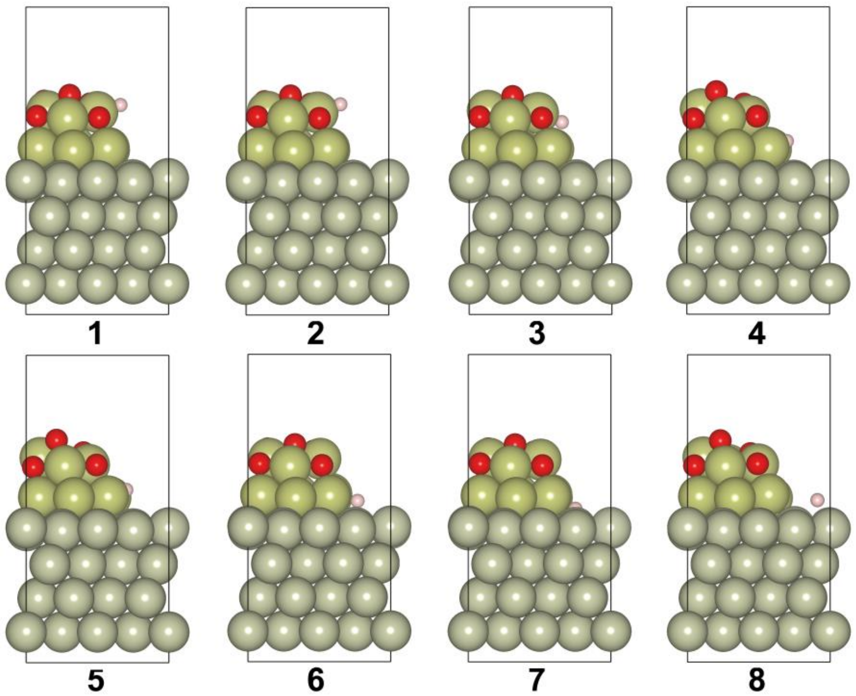

Figure S25. The optimized configurations of hydrogen adsorption (H*) on the various site along the IrO_2 to the Ir/Rh interface.

Table S1. EXAFS fitting parameters at the Rh K-edge for various samples (S_0^2=0.6837).

<table>
<tr><th>Sample</th><th>Shell</th><th>CN^a</th><th>R(Å)^b</th><th>σ²(Å²)^c</th><th>ΔE₀(eV)^d</th><th>R factor</th></tr>
<tr><td>Ro foil</td><td>Rh-Rh</td><td>12 (set)</td><td>2.68</td><td>0.004</td><td>3.6±0.4</td><td>0.018</td></tr>
<tr><td rowspan="2">Ir@Rhene</td><td>Rh-Rh</td><td>9.2±0.5</td><td>2.67</td><td>0.003</td><td rowspan="2">-6.5±0.7</td><td rowspan="2">0.013</td></tr>
<tr><td>Rh-Ir</td><td>1.0±0.7</td><td>2.68</td><td>0.001</td></tr>
<tr><td>Rhene</td><td>Rh-Rh</td><td>10.1±0.5</td><td>2.71</td><td>0.003</td><td>-5.9±0.5</td><td>0.018</td></tr>
<tr><td rowspan="2">Rh₂O₃</td><td>Rh-O</td><td>7.4±0.4</td><td>2.05</td><td>0.003</td><td rowspan="2">2.0±0.5</td><td rowspan="2">0.017</td></tr>
<tr><td>Rh-Rh</td><td>3.5±0.5</td><td>3.03</td><td>0.003</td></tr>
</table>

Note: [a]*CN*: coordination numbers; [b]*R*: bond distance; [c]σ^2: Debye-Waller factors; ΔE_0: the inner potential correction; *R* factor: goodness of fit.

Table S2. EXAFS fitting parameters at the Ir L_3-edge for various samples. (S_0^2=0.6905).

**Sample**	**Shell**	***CN***^{a}	**R(Å)**^{b}	**σ^2(Å^2)**^{c}	**ΔE_0(eV)**^{d}	***R* factor**
Ir foil	Ir-Ir	12 (set)	2.71	0.003	8.1±0.5	0.019
Ir@Rhene	Ir-O	2.7±0.2	1.99	0.006	9.3±0.5	0.009
	Ir-Ir	7.0±0.5	2.74	0.005		
	Ir-Rh	0.7±0.6	2.83	0.008		
IrO_2	Ir-O	6.8±0.4	1.97	0.002	7.9±0.6	0.018
	Ir-Ir	13.8±2.5	3.54	0.004		
	Ir-O	7.8±2.9	3.57	0.002		

Note: ^{a}*CN*: coordination numbers; ^{b}*R*: bond distance; $^c\sigma^2$: Debye-Waller factors; ΔE_0: the inner potential correction; *R* factor: goodness of fit.

Table S3. Summary of the actual amount of catalyst loading. The Pt, Ir, and Rh contents were determined using ICP-MS analysis.

Catalysts	Loading amount in WE[a]	Loading amount in WE[a]
Pt/C	1.78 μg	25.2 μg cm^{-2}
Ir/C	1.58 μg	22.4 μg cm^{-2}
Rhene/C	1.27 μg	18.0 μg cm^{-2}
Ir@Rhene/C	0.96 μg (Rh 0.60 μg + Ir 0.36 μg)	13.6 μg cm^{-2}

Note: [a]WE: working electrode.

Table S4. Summary of HER properties of the most recent reported high-performance Rh-based catalysts (in 1 M KOH solution).

**Catalysts**	**Loading (mg cm^{-2})**	**Overpotential (mV)**	**Tafel slope (mV dec^{-1})**	**Mass activity (A mg^{-1})**	**Ref.**
Ir@Rhene/C	0.014	17	14.7	4.61	This work
MoO_x-Rh/C	0.023	15	16	2.32	[S1]
HCP Rh NSs	0.017	37.8	98.3	0.82	[S2]
$RhSe_2$	0.140	49.9	39	0.04	[S3]
Rh/RhO_2	0.04	14	30	1.30	[S4]
RhPdH/C	0.015	40	35.7	1.16	[S5]
$RuRh_2$ NSs/C	0.190	24	31	0.15	[S6]
Rh/SWNTs	0.009	48	27	1.20	[S7]
Rh_2P/C	0.016	30	50	1.53	[S8]
Rh NSs	0.015	43	107.2	0.81	[S9]

Table S5. Comparison of the HER activity of the as-prepared Ir@Rhene with other state-of-the-art PGMs-based catalysts reported before (in 1 M KOH solution).

**Catalysts**	**η_{10} for HER (mV)**	**Tafel slope (mV dec^{-1})**	**Ref**
Ir@Rhene/C	17	14.7	This work
Pt@CoS_2/CC	24	82	[S10]
RuP_2@NPC	52	69	[S11]
Au-Ru-2 NWs	50	30.8	[S12]
Ni_3N/Pt	50	36.5	[S13]
Ir-NR/C	42	35.2	[S14]
N-PdIr	34	81.9	[S15]
Mo_2C@NC@Pt	47	57	[S16]
IrRh NAs	35	48.4	[S17]
Ru-H	16.4	28.6	[S18]
Ru@C_2N	17	38	[S19]
RhRu-MPSs	25	47.5	[S20]
Ru_1/NC	114	117	[S21]
IrPdH NDs/C	25	47.2	[S22]
WC@C@Pt	34	27	[S23]
PtSA/NC-DG	41	40	[S24]
Pt-SAs/$MoSe_2$	29	34	[S25]
Pt@PCM	139	73.6	[S26]
Pt_{doped}@MoC_x	32	33	[S27]
RhCu NWs-2	78	118	[S28]

References

[S1]. J. Wu, J. Fan, X. Zhao, Y. Wang, D. Wang, H. Liu, L. Gu, Q. Zhang, L. Zheng, X. Cui, D. J. Singh, W. Zheng, *Angew. Chem. Int. Ed.* **2022**, 61, e202207512.

[S2]. Z. Zhang, G. Liu, X. Cui, Y. Gong, D. Yi, Q. Zhang, C. Zhu, F. Saleem, B. Chen, Z. Lai, Q. Yun, H. Cheng, Z. Huang, Y. Peng, Z. Fan, B. Li, W. Dai, W. Chen, Y. Du, L. Ma, C.

J. Sun, I. Hwang, S. Chen, L. Song, F. Ding, L. Gu, Y. Zhu, H. Zhang, *Sci. Adv.* **2021**, 7, eabd6647.

[S3]. W. Zhong, B. Xiao, Z. Lin, Z. Wang, L. Huang, S. Shen, Q. Zhang, L. Gu, *Adv. Mater.* **2021**, *33*, e2007894.

[S4]. Z. Li, Y. Feng, Y. L. Liang, C. Q. Cheng, C. K. Dong, H. Liu, X. W. Du, *Adv. Mater.* **2020**, *32*, e1908521.

[S5]. J. Fan, J. Wu, X. Cui, L. Gu, Q. Zhang, F. Meng, B. H. Lei, D. J. Singh, W. Zheng, *J. Am. Chem. Soc.* **2020**, *142*, 3645-3651.

[S6]. X. Mu, J. Gu, F. Feng, Z. Xiao, C. Chen, S. Liu, S. Mu, *Adv. Sci.* **2021**, *8*, 2002341.

[S7]. W. Q. Zhang, X. Zhang, L. Chen, J. Y. Dai, Y. Ding, L. F. Ji, J. Zhao, M. Yan, F. C. Yang, C. R. Chang, S. J. Guo, *ACS Catal.* **2018**, *8*, 8092-8099.

[S8]. F. L. Yang, Y. M. Zhao, Y. S. Du, Y. T. Chen, G. Z. Cheng, S. L. Chen, W. Luo, *Adv. Energy Mater.* **2018**, *8*, 1703489.

[S9]. N. Zhang, Q. Shao, Y. Pi, J. Guo, X. Huang, *Chem. Mater.* **2017**, 29, 5009-5015.

[S10]. X. P. Han, X. Y. Wu, Y. D. Deng, J. Liu, J. Lu, C. Zhang, W. B. Hu, *Adv. Energy Mater.* **2018**, 8, 1800935.

[S11]. Z. H. Pu, I. Amiinu, Z. K. Kou, W. Q. Li, S. C. Mu, *Angew. Chem. Int. Ed.* **2017**, 56, 11559-11564.

[S12]. Q. P. Lu, A. L. Wang, Y. Gong, W. Hao, H. F. Cheng, J. Z. Chen, B. Li, N. L. Yang, W. X. Niu, J. Wang, Y. F. Yu, X. Zhang, Y. Chen, Z. X. Fan, X. J. Wu, J. P. Chen, J. Luo, S. Z. Li, L. Gu, H. Zhang, *Nat. Chem.* **2018**, 10, 456-461.

[S13]. Y. H. Wang, L. Chen, X. M. Yu, Y. G. Wang, G. F. Zheng, *Adv. Energy Mater.* **2017**, 7, 1601390.

[S14]. F. Luo, L. Guo, Y. H. Xie, J. X. Xu, K. G. Qu, Z. H. Yang, *Appl. Catal. B: Environ.* **2020**, 279, 119394.

[S15]. Q. Mao, K. Deng, W. Wang, P. Wang, Y. Xu, Z. Wang, X. Li, L. Wang, H. Wang, *J. Mater. Chem. A* **2022**, 10, 8364-8370.

[S16]. J. Q. Chi, J. Y. Xie, W. W. Zhang, B. Dong, J. F. Qin, X. Y. Zhang, J. H. Lin, Y. M. Chai, C. G. Liu, *ACS Appl. Mater. Interfaces* **2019**, 11, 4047–4056.

[S17]. C. Li, Y. Xu, S. Liu, S. Yin, H. Yu, Z. Wang, X. Li, L. Wang, H. Wang, *ACS Sustainable*

Chem. Eng. **2019**, 7, 18, 15747–15754.

[S18].J. Xu, X. Kong, *Small methods* **2022**, 6, 2101432.

[S19].J. Mahmood, F. Li, S. M. Jung, M. S. Okyay, I. Ahmad, S. J. Kim, N. Park, H. Y. Jeong, J. B. Baek, *Nat. Nanotechnol.* **2017**, 12, 441-446.

[S20].Y. Li, Y. Guo, S. Yang, Q. Li, S. Chen, B. Lu, H. Zou, X. Liu, X. Tong, H. Yang, *ACS Appl. Mater. Interfaces* **2021**, 13, 5052–5060.

[S21].Q. Liang, Q. Li, L. Xie, H. Zeng, S. Zhou, Y. Huang, M. Yan, X. Zhang, T. Liu, J. Zeng, K. Liang, O. Terasaki, D. Zhao, L. Jiang, B. Kong, *ACS Nano* **2022**, 16, 7993–8004.

[S22].D. Wang, X. Jiang, Z. Lin, X. Zeng, Y. Zhu, Y. Wang, M. Gong, Y. Tang, G. Fu, *Small*, **2022**, 18, 2204063

[S23].Z. W. Liu, X. T. Huo, K. Xi, P. Li, L. N. Yue, M. Huang, G. Q. Suo, L. Xu, W. Wang, X. H. Qu, *Energy Stor. Mater*. **2018**, 10, 268-274.

[S24].Y. H. Zhu, P. F. Tian, H. L. Jiang, J. R. Mu, L. Meng, X. Z. Su, Y. Wang, Y. X. Lin, Y. H. Zhu, L. Song, C. Z. Li, *CCS Chem*. **2020**, 2, 2539-2547.

[S25].Y. Shi, Z. R. Ma, Y. Y. Xiao, Y. C. Yin, W. M. Huang, Z. C. Huang, Y. Z. Zheng, F. Y. Mu, R. Huang, G. Y. Shi, Y. Y. Sun, X. H. Xia, W. Chen, *Nat. Commun.* **2021**, 12, 3021.

[S26].H. B. Zhang, P. F. An, W. Zhou, B. Y. Guan, P. Zhang, J. C. Dong, X. W. Lou, *Sci. Adv.* **2018**, 4, eaao6657.

[S27].T. Ma, H. Cao, S. Li, S. Cao, Z. Zhao, Z. Wu, R. Yan, C. Yang, Y. Wang, P. A. van Aken, L. Qiu, Y. G. Wang, C. Cheng, *Adv. Mater.* **2022**, e2206368.

[S28].D. Cao, H. X. Xu, D. J. Cheng, *Adv. Energy Mater.* **2020**, 10, 1903038.
